# Bioactive Naphtho-α-Pyranones from Two Endophytic Fungi of the Genus *Polyphilus*

**DOI:** 10.3390/antibiotics12081273

**Published:** 2023-08-02

**Authors:** Jan-Peer Wennrich, Ellen Sepanian, Sherif S. Ebada, Natalia A. Llanos-Lopez, Samad Ashrafi, Wolfgang Maier, Tibor Kurtán, Marc Stadler

**Affiliations:** 1 Department of Microbial Drugs, Helmholtz Centre for Infection Research (HZI), Inhoffenstrasse 7, 38124 Braunschweig, Germany; jan-peer.wennrich@helmholtz-hzi.de (J.-P.W.); ellen.sepanian@gmail.com (E.S.); natalia.llanos@helmholtz-hzi.de (N.A.L.-L.); 2 Institute of Microbiology, Technische Universität Braunschweig, Spielmannstraße 7, 38106 Braunschweig, Germany; 3 Department of Pharmacognosy, Faculty of Pharmacy, Ain Shams University, Cairo 11566, Egypt; 4 Institute for Epidemiology and Pathogen Diagnostics, Julius Kühn Institute (JKI)—Federal Research Centre for Cultivated Plants, Messeweg 11-12, 38104 Braunschweig, Germany; samad.ashrafi@julius-kuehn.de (S.A.); wolfgang.maier@julius-kuehn.de (W.M.); 5 Institute for Crop and Soil Science, Julius Kühn Institute (JKI)—Federal Research Centre for Cultivated Plants, Bundesallee 58, 38116 Braunschweig, Germany; 6 Department of Organic Chemistry, University of Debrecen, P.O. Box 400, 4002 Debrecen, Hungary; kurtan.tibor@science.unideb.hu

**Keywords:** *Polyphilus*, Helotiales, Ascomycota, naphthopyranones, antimicrobial

## Abstract

In the course of our survey to study the metabolic potential of two species of a new helotialean genus *Polyphilus*, namely *P. frankenii* and *P. sieberi*, their crude extracts were obtained using different cultivation techniques, which led to the isolation and characterization of two new naphtho-*α*-pyranone derivatives recognized as a monomer (**1**) and its 6,6′-homodimer (**2**) together with two known diketopiperazine congeners, outovirin B (**3**) and (3*S*,6*S*)-3,6-dibenzylpiperazine-2,5-dione (**4**). The structures of isolated compounds were determined based on extensive 1D and 2D NMR and HRESIMS. The absolute configuration of new naphtho-*α*-pyranones was determined using a comparison of their experimental ECD spectra with those of related structural analogues. 6,6′-binaphtho-*α*-pyranone talaroderxine C (**2**) exhibited potent cytotoxic activity against different mammalian cell lines with IC_50_ values in the low micromolar to nanomolar range. In addition, talaroderxine C unveiled stronger antimicrobial activity against *Bacillus subtilis* rather than *Staphylococcus aureus* with MIC values of 0.52 µg mL^−1^ (0.83 µM) compared to 66.6 µg mL^−1^ (105.70 µM), respectively.

## 1. Introduction

Naphtho-*α*-pyranones comprise a unique class of fungal metabolites that have been reported as monomers, such as semiviriditoxin [1,2,3], semivioxanthin [1,2,3], and penicitor A [4] and its 7-*O*-methyl derivative [5]. In addition, naptho-*α*-pyranone dimers have been firstly reported as “mycotoxins”, such as viriditoxin [6] followed by several other symmetric dimers featuring either 6,6′-linkages, such as asteromine [7], talaroderxines [8], pigmentosins [9,10], and aschernaphtopyrone A [11], or 8,8′-linkages, such as vioxanthin [12,13], mycopyranone [14], aschernaphtopyrone B [11], and lichenocholin A [15].

A rare 5,8′-linkage was also recently reported in lulworthinone obtained from a marine-derived fungus: *Lulworthia medusa* [16]. Several members of the dimer class have been reported to exhibit potential antibacterial activity. In particular, viriditioxin has been reported to inhibit FtsZ, which is essential for bacterial cell division [17]. Based on the fact that antibiotic resistance is an exacerbating problem, the need for new antimicrobial agents remains an unmet demand.

**Figure 1:**
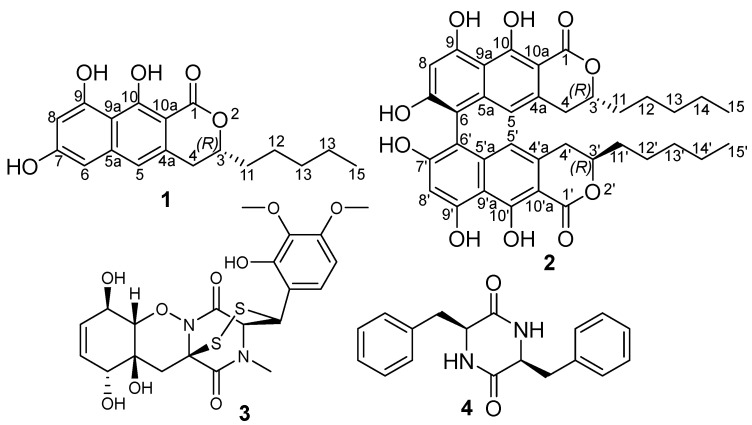
Chemical structures of **1**–**4**.

During our ongoing research targeting the discovery of new fungal metabolites with potential antimicrobial activities, in this study, we investigated the root endophytic representatives of the recently described fungal genus *Polyphilus*, namely *P. frankenii* and *P. sieberi* [18]. The chemical exploration resulted in the isolation and identification of a monomer (**1**) and its symmetric 6,6′-homodimer (**2**) (Figure 1) together with two known metabolites identified as outovirin B (**3**) [19] and (3*S*,6*S*)-3,6-dibenzylpiperazine-2,5-dione (**4**) [20]. In this study, we report the isolation and structure elucidation of two new naphto-*α*-pyranone derivatives together with their antimicrobial and cytotoxic activity, which were recorded in our routine assays.

## 2. Results and Discussion

### 2.1. Isolation and Identification of Compounds (***1*** and ***2***)

**Figure 2:**
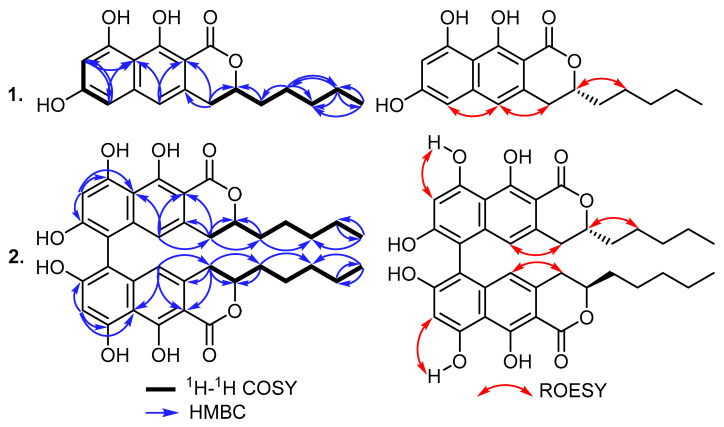
Key COSY, HMBC, and ROESY correlations of **1** and **2**.

Compound **1** was purified as a white solid powder, and its molecular formula was established to be C_18_H_20_O_5_ based on its HRESIMS spectrum, which revealed pseudomolecular ion peaks at *m*/*z* 317.1386 [M+H]^+^ (calculated for 317.1384) and at *m*/*z* 339.1202 [M+Na]^+^ (calculated for 339.1203), indicating the existence of nine degrees of unsaturation. The ^13^C NMR spectral data of **1** (Table 1) displayed the presence of fifteen carbon resonances that can be differentiated into eight quaternary carbon atoms recognized as one carbonyl at *δ*_C_ 170.7 (C-1) and seven olefinic (two oxygenated) carbon atoms at *δ*_C_ 162.6 (C-10), 161.0 (C-7), 160.7 (C-9), 140.6 (C-5a), 133.9 (C-4a), 107.2 (C-9a), and 98.8 (C-10a). In addition, the ^13^C NMR spectrum of **1** also revealed the presence of four tertiary (one aliphatic and three olefinic) carbon atoms at *δ*_C_ 114.5 (C-5), 101.5 (C-8), 101.4 (C-6), and 79.3 (C-3) along with four methylenes (*δ*_C_ 34.0 (C-11), 32.3 (C-4), 31.0 (C-13), and 22.0 (C-14)) and one methyl carbon atom (*δ*_C_ 13.7 (C-15)). By comparing the obtained results with the reported literature, compound **1** was suggested to be a naphthopyrone derivative related to those reported as fungal metabolites, such as penicitor A [4,5], semiviriditoxin [1,2], and semivioxanthin [3]. Further structural features of **1** were concluded using 2D NMR spectra including ^1^H-^1^H COSY, HMBC, and HSQC. The ^1^H-^1^H COSY spectrum of **1** revealed two main spin systems, with one extending over two *meta*-positioned aromatic protons at *δ*_H_ 6.31 (H-6) and 6.47 (H-8) while the second spin system was found to begin at a methylene group at *δ*_H_ 2.88/*δ*_H_ 3.00 (H_2_-4) and extended to an aliphatic oxygenated methine group at *δ*_H_ 4.58 (H-3), thus extending over four methylenes, forming an aliphatic side chain at *δ*_H_ 1.68/*δ*_H_ 1.74 (H_2_-11), *δ*_H_ 1.41/*δ*_H_ 1.46 (H_2_-12), and *δ*_H_ 1.31 (H_2_-14 and H_2_-13), and ending with a terminal triplet methyl group at *δ*_H_ 0.88 (H_3_-15) to confirm the presence of the *n*-pentyl side chain. The HMBC spectrum of **1** (Figure 2) revealed key correlations from H_2_-4 to four carbon atoms ascribed to C-10a (*δ*_C_ 98.8), C-4a (*δ*_C_ 133.9), C-3 (*δ*_C_ 79.3), and C-11 (*δ*_C_ 34.0), whereas additional HMBC correlations from H_2_-11 to C-3 confirmed that the *n*-pentyl side chain was present at C-3. Further key HMBC correlations (Figure 2) were also observed in the aromatic protons from H-5, H-6, and H-8 to C-9a (*δ*_C_ 107.2) and from H-5 and H_2_-4 to C-10a, which confirmed the depicted structure of **1** as a naphthopyrone derivative. The ECD spectrum of **1** (see Supplementary Materials: Figure S9) showed negative Cotton effects (CEs) at 219 nm and 267 nm, a weaker positive one at 242 nm, and a broad positive plateau within the range of 280-400. This ECD spectrum was a near mirror image of that of (*S*)-7-*O*-methylpenicitor A [5], which differed only in the C-9 and C-14 methoxy groups, and its absolute configuration (AC) was determined using ECD calculations and single-crystal X-ray diffraction analysis. Due to the mirror image ECD curves, the AC of **1** was assigned as (*R*), and as a new naphthopyrone derivative, it was given the trivial name semitalaroderxine C.

Compound **2** was isolated as a white solid powder that revealed in a pseudomolecular ion peak at *m*/*z* 631.2541 [M+H]^+^ (calculated for 631.2538) its HRESIMS spectrum, confirming its molecular formula as C_36_H_38_O_10_ and indicating its inclusion of eighteen degrees of unsaturation. Intriguingly, by comparing the molecular formulas of **1** and **2**, it could be obviously observed that **2** is a symmetric dimer of the two monomers of **1**. This assumption was further confirmed using ^13^C NMR spectral data (Table 1), which revealed only eighteen carbon resonances; thus, each was assigned to two electromagnetically equivalent carbon atoms in the two monomers. The ^1^H NMR spectral data of **2** (Table 1) displayed an identical set of proton resonances to those of **1,** except in the absence of one aromatic proton at *δ*_H_ 6.31 (H-6) in **1** and hence suggesting that compound **2** is a 6,6′-binaphthopyrone dimer of semitalaroderxine C (**1**). Based on the obtained results and by searching the reported literature, compound **2** was found to be related to the previously reported 6,6′-binaphthopyrone dimers, talaroderxines A and B, that were reported from a soil-derived fungus *Talaromyces derxii* [8] and pigmentosins A/B [10,21].

The major structural difference between **2** and talaroderxines A/B was the presence of *n*-pentyl in **2** instead of the *n*-propyl side chain in talaroderxines A/B. Since compound **2** is the 6,6′-linked axially chiral homodimer of **1**, the (3*S*,3′*S*) absolute configuration of the central chirality elements was deduced on the basis of their common biosynthetic origin. The (a*S*) axial chirality of **2**, arising from the hindered rotation around the C-6-C-6′ biaryl axis, was determined by comparing its experimental ECD spectrum (see Supplementary Materials Figure S18) with those of related 6,6′-linked *bis*-naphthopyrone pigmentosins A and B [10] and talaroderxine A [8]. Compound **2** showed an intense positive exciton-coupled couplet centered at 260 nm (268 nm (Δε: +16.58); 252 nm (Δε: −14.15)), which was a mirror image of the negative couplet of (a*R*)-pigmentosins A and B and (a*R*)-talaroderxine B [8,9,10]. Compound **2** was identified as a new 6,6′-linked *bis*-naphthopyrone homodimer with (a*S*) axial chirality, which was named talaroderxine C.

### 2.2. Biological Assays

Due to the limited amounts of compounds **1**, **3**, and **4**, only talaroderxine C (**2**) was subjected to antimicrobial and cytotoxicity assays. Against all tested microorganisms, talaroderxine C disclosed potent antimicrobial activity against *Bacillus subtilis* with a minimal inhibitory concentration (MIC) of 0.52 µg/mL (0.83 µM). In the case of *Staphylococcus aureus*, it revealed moderate activity with an MIC of 66.6 µg/mL (105.70 µM). In addition, the obtained results of the cytotoxicity (3-(4,5-dimethylthiayol-2-yl)-2,5-diphenyltetrazolium bromide, MTT) assay (Table 2) revealed that talaroderxine C (**2**) exhibits pan-cytotoxic activity against all tested cell lines, with IC_50_ values falling within the low micromolar to nanomolar range. In particular, against breast adenocarcinoma (MCF-7) cells, talaroderxine C revealed a high potency (IC_50_ = 68 nM). Nevertheless, outovirin B (**3**) is structurally recognized as a gliovirin-like compound [22] that was reported to exhibit selective antifungal and anti-inflammatory [23] and antitrypanosomal [24] and antimycobacterial [25] activities.

## 3. Materials and Methods

### 3.1. General Experimental Procedures

UV measurements were performed using a Shimadzu UV-VIS spectrophotometer UV-2450 (Shimadzu^®^, Kyoto, Japan), and ECD spectra were obtained using a Jasco J-815 spectropolarimeter (JASCO^®^, Pfungstadt, Germany). Optical rotation values were measured using a PerkinElmer 241 polarimeter at 20 °C (Anton-Paar Opto Tec GmbH, Seelze, Germany). High-resolution electrospray ionization mass spectra (HR-ESI-MS) were acquired using an Agilent 1200 Infinity Series HPLC-UV system (Agilent Technologies^®^, Santa Clara, CA, USA), utilizing a C_18_ Acquity UPLC BEH column (2.1 × 50 mm, 1.7 µm: Waters, Milford, MA, USA); solvent A: H_2_O + 0.1% formic acid; solvent B: acetonitrile (MeCN) + 0.1% formic acid; gradient: 5% B for 0.5 min increasing to 100% B in 19.5 min and maintaining 100% B for 5 min at a flow rate of 0.6 mL min^−1^ (UV/Vis detection 190–600 nm) connected to a time-of-flight mass spectrometer (ESI-TOF-MS, Maxis, Bruker, Billerica, MA, USA) (scan range: 100–2500 *m*/*z*; rate 2: Hz; capillary voltage: 4500 V; dry temperature: 200 °C). NMR spectra were recorded using an Avance III 500 spectrometer (Bruker^®^, Billerica, MA, USA; ^1^H-NMR: 500 MHz; ^13^C-NMR: 125 MHz), dissolving compounds in deuterated methanol-*d*_4_ and deuterated DMSO-*d*_6_.

### 3.2. Fermentation, Extraction, and Isolation

The two fungal species explored in this study namely, *P. frankenii* strain V16 (DSM 106521) and *P. sieberi* strain Ref052 (DSM 106515) were recognized as endophytic fungi associated with the roots of *Pinus sylvetris* and *Asclepias syriaca* collected from Villerupt (France) and Bugac (Hungary), respectively. The fungi were maintained on a YM6.3 agar (D-glucose: 4 g L^−1^; malt extract: 10 g L^−1^: yeast extract: 4 g L^−1^; agar: 20 g L^−1^; pH adjusted to 6.3 and sterilized by autoclaving) at 23 °C. To prepare seed cultures for the following larger-scale cultivation, five mycelia plugs measuring 25 mm^2^ were transferred to a 500 mL Erlenmeyer shaking flask containing 200 mL of Q6/2 media (D-glucose: 2.5 g L^−1^; glycerine: 10 g L^−1^; cotton seed flour: 5 g L^−1^; pH: 7.2) and incubated at 23 °C and 140 min^−1^. After achieving a sufficient amount of biomass, the culture broth was homogenized using Ultra-Turrax (T25 easy clean digital, IKA) equipped with an S25 N-25F dispersing tool at 10,000 rpm for 10 s. This seed culture was used for all following cultivations as an inoculum for YM6.3, BRFT, and WOFT media (K_2_HPO_4_: 0.5 g L^−1^; sodium tartrate: 0.5 g L^−1^; yeast extract: 1 g L^−1^; 100 mL of solution was added to 28 g brown rice or whole oat and autoclaved).

#### 3.2.1. Solid State Fermentation

Six Erlenmeyer culture flasks with BRFT and WOFT were inoculated with 6 mL of homogenized seed culture, mixed under sterile conditions, and incubated at room temperature for two, three, and four weeks in the dark. After the planned incubation time, the cultures were stopped by adding 250 mL of acetone, mixed with a spatula, and kept in an ultrasonic bath for 30 min at 40 °C for extraction. The liquid was separated from the solid phase via filtration. The extraction was repeated twice using fresh acetone. The filtrate was evaporated under a vacuum at 40 °C in a rotary evaporator, and the remaining aqueous phase was filled up to 50 mL with distilled water and extracted with EtOAc (1:1) in a separatory funnel three times. The organic phase (EtOAc) was separated, evaporated under reduced pressure, and then dissolved again in 5 mL of MeOH and extracted again with 45 mL of heptane. Extraction was performed in a separatory funnel twice with fresh heptane. Both fractions (heptane and methanol) were evaporated to dryness.

#### 3.2.2. Liquid Fermentation

In this study, 0.5% of the homogenized inoculum was transferred into twelve 2 L Erlenmeyer culture flasks containing 400 mL of YM6.3 medium. The content of glucose was monitored using test stripes (Medi-Test Glucose, Machery-Nagel^®^, Düren, Germany). Incubation was terminated after five days of glucose depletion. Mycelia and supernatant were separated using a Büchner funnel and a vacuum pump. The extraction of metabolites of the mycelia followed the procedure of solid-state fermentation. The culture broth was mixed with 2% AmberLite^TM^ XAD^TM^ 16N polymeric absorbent and stirred for 4 h on a magnetic stirrer. The extraction of metabolites was carried out with acetone under stirring. Afterward, the purification scheme followed the same steps as described above for solid-state extraction.

#### 3.2.3. Analytical HPLC

The extracts obtained were dissolved in Acetone:MeOH (1:1) and adjusted to a concentration of 4.5 mg mL^−1^. An injection volume of 2 µL was applied to an UltiMate^®^ 3000 Series uHPLC (Thermo Fisher Scientific^®^, Waltman, MA, USA). Mass spectrometry was performed with a connected amaZon^®^ speed ESI Iontrap MS (Amazon, Bruker). HRESIMS measurements were performed with sample concentrations of 1 mg mL^−1^ with an Agilent 1200 series HPLC-UV system in combination with an ESI-TOF-MS (Maxis, Bruker). The conditions were identical to the methods described before [26].

#### 3.2.4. Isolation of Compounds

The mycelial extract of *Polyphilus sieberi* Ref052 (DSM 106515) cultivated in YM6.3 media (86 mg) was separated with a Reveleris^®^ X2 flash chromatography system using a FlashPure ID Silica 12 g cartridge. The mobile phase consisted of three solvent mixtures supplemented with 0.1% formic acid: solvent A (heptane 100%), solvent B (heptane 58%, TBME 40%, and MeOH 2%), and solvent C (Acetone 37.5%, DCM 37.5%, and MeOH 25%); flow rate: 30 mL min^−1^; gradient: 3 min B at 0%, increasing to 100% B in 10 min, maintaining 100% B for 5 min, switching to solvent mixture B and C, starting from 0% C and increasing to 100% C in 10 min, and maintaining 100% C for 10 min. All the following flash chromatographic separations were performed, implementing the same conditions. The separation led to the purification of **1** (1.1 mg; t_R_ = 8.5 min).

The second *Polyphilus frankenii* V16 (DSM 106521) strain was cultivated in BRFT and WOFT media. Due to the high similarity observed in ESI-MS profiles for 2–4 weeks, the extracts were combined to yield a crude extract (672 mg) and subjected to the Reveleris^©^ X2 equipped with a FlashPure ID Silica 24 g cartridge. Further purification steps of the resulting fraction 5 (49.2 mg, t_R_ = 13.5 min) were carried out on a Büchi Pure C-850 FlashPrep equipped with a Gemini C18 (250 × 21.2 mm, 10 µm; Phenomenex) (solvent A: H_2_O + 0.1% formic acid; solvent B: MeCN + 0.1% formic acid; flow rate: 20 mL min^−1^; gradient: 5 min maintaining 40% B, increasing B to 90% in 50 min, increasing to 100% B in 5 min, and maintaining 100% B for 10 min) led to the isolation of **2** (15.8 mg, t_R_ = 53.4 min). The mycelial extract of the cultivation in YM6.3 media was weighed at 35 mg, and it was purified on a Gilson PLC 2250 equipped with a Gemini C18 (250 × 21.2 mm, 10 µm; Phenomenex, Torrance, CA, USA) (solvent A: H_2_O + 0.1% formic acid; solvent B: MeCN + 0.1% formic acid; flow rate: 20 mL min^−1^; gradient: 10 min maintaining 5% B, increasing B to 50% in 45 min, increasing to 100% B in 10 min, and maintaining 100% B for 10 min. This led to the isolation of known metabolites **3** (0.2 mg; t_R_ = 17.3 min) and **4** (0.2 mg; t_R_ = 27.5 min).

3.2.1 Semitalaroderxine C (**1**): white solid powder; 0.59 mg; ${\left[\alpha \right]}_{D}^{20}$ –7.5 (*c* 0.04, chloroform); UV/Vis (MeOH): λ_max_ (log *ε*) = 193.5 (0.8), 200.0 (0.9), 220.5 (1.0), 261.0 (2.7), 314.5 (0.2), 369.5 (0.6) nm; ECD (*c* = 3.16 × 10^−4^ M; MeOH) λ [nm], (Δε) 373 (1.4), 310 (1.0), 268 (−3.2), 243 (1.1), 218 (−4.6), 196 (4.2); NMR data (^1^H NMR: 500 MHz, ^13^C NMR: 125 MHz in DMSO-*d*_6_) see Table 1; HR-(+)ESIMS: *m*/*z* 299.1277 [M–H_2_O+H]^+^ (calcd. 299.1278 for C_18_H_19_O_4_^+^), *m*/*z* 317.1386 [M+H]^+^ (calcd. 317.1384 for C_18_H_21_O_5_^+^), 339.1203 [M+Na]^+^ (calcd. 339.1203 for C_18_H_20_NaO_5_^+^), 655.2516 [2M+Na]^+^ (calcd. 655.2514 for C_36_H_40_NaO_10_^+^).

3.2.2 Talaroderxine C (**2**): white solid powder; 3.49 mg; ${\left[\alpha \right]}_{D}^{20}$ +57.5 (*c* 1.0, DMSO-*d*_6_); UV/Vis (MeOH): λ_max_ (log *ε*) = 223.0 (0.1), 265.5 (0.1), 376.5 (0.04) nm; ECD (*c* = 3.17 × 10^−4^ M; MeOH) λ [nm], (Δε) 268 (82.9), 252 (−70.7); NMR data (^1^H NMR: 500 MHz, ^13^C NMR: 125 MHz in DMSO-*d*_6_) see Table 1; HR-(+)ESIMS: *m*/*z* 631.2541 [M+H]^+^ (calcd. 631.2538 for C_36_H_39_O_10_^+^).

### 3.3. Antimicrobial Assay

Due to the limited amounts of compounds **1**, **3**, and **4**, only talaroderxine C (**2**) was assessed for their antimicrobial activity based on their minimum inhibitory concentration (MIC), following our previously reported protocol [26], against five pathogenic fungi, including *Candida albicans* DSM 1665, *Mucor hiemails* DSM 2656, *Pichia anomala* DSM 6766, *Rhodotorula glutinis* DSM 10134, and *Schizosaccharomyces pombe* DSM 70572; three Gram-positive bacteria, including *Bacillus subtilis* DSM 10, *Mycobacterium smegmatis* DSM ATCC 700084, and *Staphylococcus aureus* DSM 436; and four Gram-negative bacteria, including *Chromobacterium violaceum* DSM 30191, *Acinetobacter baumanii* DSM 30008, *Escherichia coli* DSM 1116, and *Pseudomonas aeruginosa* DSM PA14. Nystatin was used as an antifungal positive control, whereas oxytetracycline, ciprofloxacin, gentamycin, and kanamycin were used as positive controls against Gram-positive and Gram-negative bacteria.

### 3.4. Cytotoxicity Assay

The cytotoxicity (IC_50_) of Talaroderxine C (**2**) was also tested using the MTT (3-(4,5-dimethylthiayol-2-yl)-2,5-diphenyltetrazolium bromide) test, following the experimental procedure described by Charria-Girón et al. [27]. These compounds were tested against seven mammalian cell lines, including human endocervical adenocarcinoma KB 3.1, breast cancer MCF-7, lung cancer A549, ovary cancer SK-OV-3, prostate cancer PC-3, squamous cancer A431, and mouse fibroblasts L929. Epothilone B was used as the positive control.

## 4. Conclusions

This represents the first study of secondary metabolites from the fungal genus *Polyphilus*. In summary, semitalaroderxine C (**1**), talaroderxine C (**2**), outovirin B (**3**), and (3*S*,6*S*)-3,6-dibenzylpiperazine-2,5-dione (**4**) could be isolated and elucidated where **1** and **2** are recognized among naphtha-*α*-pyranone congeners that are well-known for a vast array of bioactivities. Talaroderxine C showed potent antimicrobial activity against *Bacillus subtilis* with an MIC of 0.52 µg/mL. In addition, it exhibited pan-cytotoxic activity against all tested cell lines, with IC_50_ values falling in the low micromolar to nanomolar range. Talaroderxine C (**2**) showed high potency (IC_50_ = 68 nM), particularly against breast adenocarcinoma (MCF-7) cells. Due to the limited amounts of **3** and **4**, antimicrobial and cytotoxic activity assays were not determined, but the gliovirin-like compound [22], outovirin B (**3**), was reported to exhibit selective antifungal and anti-inflammatory [23] along with antitrypanosomal [24] and antimycobacterial [25] activities.

The strains of the genus *Polyphilus* arose from an attempt to find nematode antagonistic fungi that can be used as ecologically friendly alternatives to toxic chemicals and soil fumigants. Such biocontrol agents may turn out to be a valid alternative, especially as they are supposed to be host-specific and do not kill harmless organisms in the soil. However, the results of the current study, which showed that *Polyphilus* strains can produce rather toxic secondary metabolites, should give rise to deprioritizing them over other strains that were encountered concurrently and that were devoid of toxic metabolites. Mycopesticides, which are mostly used against insects, already have an annual market share of ca. USD 550 million [28], and this is bound to increase further in the future. Nematode antagonists will probably obtain their share as they are one of the only environmentally friendly alternatives to chemical pesticides.

## Figures and Tables

**Table 1:** ^1^H and ^13^C NMR data of **1** and **2**.

	1			2	
Pos.	*δ*_H_ (Multi, *J*(Hz)) ^a^	*δ*_C_, Type ^b,c^	Pos.	*δ*_H_ (Multi, *J*(Hz)) ^a^	*δ*_C_, Type ^b,c^
1		170.7, CO	1/1′		170.6, CO
3	4.58, m	79.3, CH	3/3′	4.50, m	79.4, CH
4	*α* 2.88, dd, (15.6, 11.1) *β* 3.00, br d, (15.6)	32.3, CH_2_	4/4′	*α* 2.72, dd, (16.2, 11.0) *β* 2.81, dd, (16.5, 3.1)	32.5, CH_2_
4a		133.9, C	4a/4′a		133.6, C
5	6.82, s	114.5, CH	5/5′	6.20, s	112.7, CH
5a		140.6, C	5a/5′a		139.9, C
6	6.31, s	101.4, CH	6/6′		107.7, C
7		161.0, C, C	7/7′		157.8, C
8	6.47, s	101.5, CH	8/8′	6.57, s	101.6, CH
9		160.7, C	9/9′		158.9, C
9a		107.2, C	9a/9′a		107.7, C
10		162.6, C	10/10′		163.2, C
10a		98.8, C	10a/10′a		98.7, C
11	*α* 1.68, m *β* 1.74, m	34.0, CH_2_	11/11′	*α* 1.57, ddd, (14.2, 10.8, 5.6) *β* 1.66, m	34.1, CH_2_
12	*α* 1.41, m; *β* 1.46, m	24.0, CH_2_	12/12′	1.36, m (2H)	23.9, CH_2_
13	1.31, m, 2H	31.0, CH_2_	13/13′	1.23, m (2H)	31.0, CH_2_
14	1.31, m, 2H	22.0, CH_2_	14/14′	1.25, m (2H)	22.0, CH_2_
15	0.88, t, (7.0)	13.9, CH_3_	15/15′	0.84, t (6.8, 3H)	13.9, CH_3_
7-O*H*	10.11, br s		7-O*H*	10.17, br s	
9-O*H*	9.66, br s		9-O*H*	9.65, s	
10-O*H*	13.35, br s		10-O*H*	13.43, br s	

Measured in DMSO-*d_6_* ^a^ at 500 MHz/^b^ at 125 MHz. ^c^ Assigned based on HMBC and HSQC spectra.

**Table 2:** Cytotoxic (IC_50_ in µM) activity results of talaroderxine C (**2**).

Compound	IC_50_					
	L929	KB3.1	A431	A549	PC-3	MCF-7
Talaroderxine C (**2**) in µM	1.19	10.32	2.38	2.06	8.73	0.068
Epothilone B in nM	0.65	0.17	0.065	0.053	0.091	0.075

## Data Availability

All data are available in the manuscript or the SI.

## Supplementary Materials

The following supporting information can be downloaded at https://www.mdpi.com/article/10.3390/antibiotics12081273/s1. Tables S1 and S2: 2D NMR data of semitalaroderxine C (**1**) and talaroderxine C (**2**); Figures S1–S4: LC-ESI-MS spectra of sole four-weeks mycelia extracts of *P. sieberi* or *P. frankenii* in different solid or liquid media; Figures S5–S14: HPLC, UV, LR-/HRESIMS, 1D/2D NMR, and ECD spectra of **1**; Figures S15–S24: HPLC, UV, LR-/HRESIMS, 1D/2D NMR, and ECD spectra of **2**; Figures S25–S30: HPLC and LR-/HRESIMS 1D/2D NMR spectra of **3**; Figures S31–S38: HPLC and LR-/HRESIMS 1D/2D NMR spectra of **4**.
