# Supplementary material for: Bioactive Naphtho-α-Pyranones from Two Endophytic Fungi of the Genus Polyphilus
Source: Antibiotics (Basel). 2023 Aug 2;12(8):1273. doi: 10.3390/antibiotics12081273 (PMC10451773; doi:10.3390/antibiotics12081273)
Supplement: Supplementary file 1 [file antibiotics-12-01273-s001.zip › antibiotics-2537628-supplementary.pdf]

Supplementary Materials for

Bioactive Naphtho-α-Pyranones from Two Endophytic Fungi of the Genus *Polyphilus*

Abstract

In the course of our survey to study the metabolic potential of two species of a new helotialean genus *Polyphilus*, namely *P. frankenii* and *P. sieberi*, their crude extracts were obtained under different cultivation techniques which led to the isolation and characterization of two new naphtho-*α*-pyranone derivatives recognized as a monomer (**1**) and its 6,6'-homodimer (**2**) together with two known diketopiperazine congeners, outovirin B (**3**) and (3*S*,6*S*)-3,6-dibenzylpiperazine-2,5-dione (**4**). Structures of isolated compounds were determined based on extensive 1D, 2D NMR and HRESIMS. The absolute configuration of new naphtho-*α*-pyranones was determined by comparison of their experimental ECD spectra with those of related structural analogues. The 6,6'-binaphtho-*α*-pyranone talaroderxine C (**2**) exhibited potent cytotoxic activity against different mammalian cell lines with IC_{50} values in the low micromolar to nanomolar range. In addition, talaroderxine C unveiled stronger antimicrobial activity against *Bacillus subtilis* rather than *Staphylococcus aureus* with MICs values of 0.52 μg mL^{-1} (0.83 μM) compared to 66.6 μg mL^{-1} (105.70 μM), respectively.

Keywords: *Polyphilus;* Helotiales; Ascomycota; naphthopyranones; antimicrobial.

Contents of Supporting Information

#	Contents	Page
1	Figure S1: LC-ESI-MS spectra of mycelia extracts of *P. sieberi* REF052 (DSM 106515) cultivated on BRFT incubated for *four* week.	S4
2	Figure S2: LC-ESI-MS spectra of methanol extracts of *P. frankenii* V16 (DSM 106521) cultivated on BRFT incubated for four week.	S4
3	Figure S3: LC-ESI-MS spectra of methanol extracts of *P. frankenii* V16 (DSM 106521) cultivated on WOFT incubated for four week.	S5
4	Figure S4: LC-ESI-MS spectra of mycelia extracts of *P. frankenii* V16 (DSM 106521) cultivated in YM6.3 media incubated for four week.	S5
5	Table S1. 2D (COSY, HMBC and ROESY) NMR correlations of **1**.	S6
6	Table S2. 2D (COSY, HMBC and ROESY) NMR correlations of **2**.	S7
7	Figure S5. LRESIMS spectra of **1**.	S8
8	Figure S6. HRESIMS spectra of **1**.	S9
9	Figure S7. UV spectrum of **1**.	S10
10	Figure S8. ^{1}H NMR spectrum of **1** in DMSO-d_6 at 500 MHz.	S11
11	Figure S9. ^{13}C NMR spectrum of **1** in DMSO-d_6 at 125 MHz.	S12
12	Figure S10. ^{1}H-^{1}H COSY spectrum of **1** in DMSO-d_6 at 500 MHz.	S13
13	Figure S11. HMBC spectrum of **1** in DMSO-d_6 at 500 MHz.	S14
14	Figure S12. HSQC spectrum of **1** in DMSO-d_6 at 500 MHz.	S15
15	Figure S13. ROESY spectrum of **1** in DMSO-d_6 at 500 MHz.	S16
16	Figure S14. Experimental ECD spectrum of **1** in methanol.	S17
17	Figure S15. LRESIMS spectra of **2**.	S18
18	Figure S16. HRESIMS spectra of **2**.	S19
19	Figure S17. UV spectrum of **2**.	S20
20	Figure S18. ^{1}H NMR spectrum of **2** in DMSO-d_6 at 500 MHz.	S21
21	Figure S19. ^{13}C NMR spectrum of **2** in DMSO-d_6 at 125 MHz.	S22
22	Figure S20. ^{1}H-^{1}H COSY spectrum of **2** in DMSO-d_6 at 500 MHz.	S23
23	Figure S21. HMBC spectrum of **2** in DMSO-d_6 at 500 MHz.	S23
24	Figure S22. HSQC spectrum of **2** in DMSO-d_6 at 500 MHz.	S24
25	Figure S23. ROESY spectrum of **2** in DMSO-d_6 at 500 MHz.	S25
26	Figure S24. Experimental ECD spectrum of **2** in methanol.	S26
27	Figure S25. LRESIMS spectra of **3**.	S27
28	Figure S26. HRESIMS spectra of **3**.	S27
29	Figure S27. ^{1}H NMR spectrum of **3** in methanol-d_4 at 500 MHz.	S28
30	Figure S28. ^{1}H-^{1}H COSY spectrum of **3** in methanol-d_4 at 500 MHz.	S29
31	Figure S29. HMBC spectrum of **3** in methanol-d_4 at 500 MHz.	S30
32	Figure S30. HSQC spectrum of **3** in methanol-d_4 at 500 MHz.	S31
33	Figure S31. LRESIMS of **4**.	S32
34	Figure S32. HRESIMS of **4**.	S32
35	Figure S33. ^{1}H NMR spectrum of **4** in DMSO-d_6 at 700 MHz.	S33
36	Figure S34. ^{13}C NMR spectrum of **4** in DMSO-d_6 at 175 MHz.	S34
37	Figure S35. ^{1}H-^{1}H COSY spectrum of **4** in DMSO-d_6 at 700 MHz.	S35
38	Figure S36. HMBC spectrum of **4** in DMSO-d_6 at 700 MHz.	S36
39	Figure S37. HSQC spectrum of **4** in DMSO-d_6 at 700 MHz.	S37
40	Figure S38. ROESY spectrum of **4** in DMSO-d_6 at 700 MHz.	S38

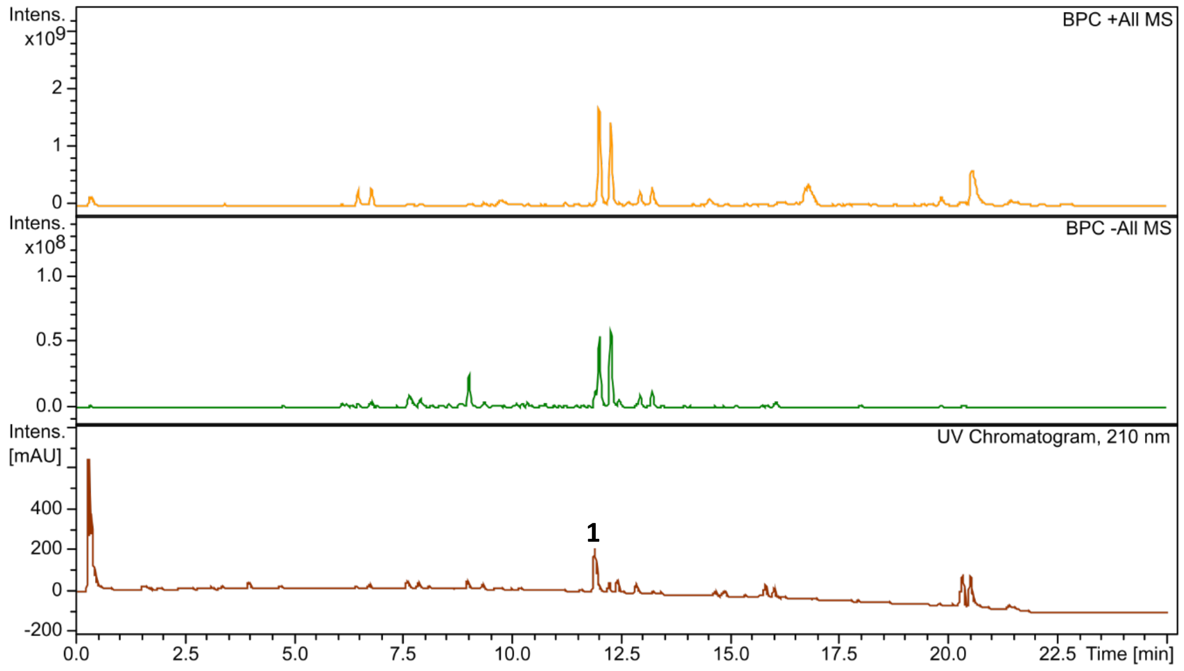

Figure S1: LC-ESI-MS spectra of mycelia extracts of *P. sieberi* REF052 (DSM 106515) cultivated on BRFT incubated for four week. Top-bottom: +MS, -MS, UV-chromatogramm at 210 nm. Numbers represents isolated semitalaroderxine C (**1**).

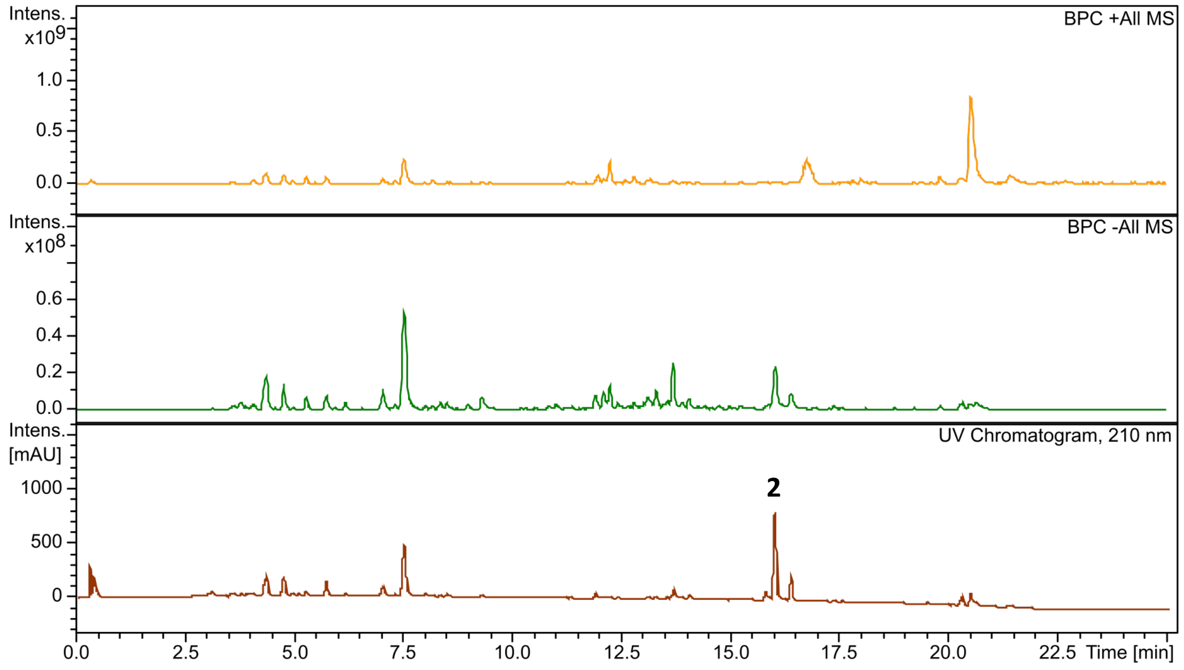

Figure S2: LC-ESI-MS spectra of methanol extracts of *P. frankenii* V16 (DSM 106521) cultivated on BRFT incubated for four week. Top-bottom: +MS, -MS, UV-chromatogramm at 210 nm. Numbers represents isolated talaroderxine C (**2**).

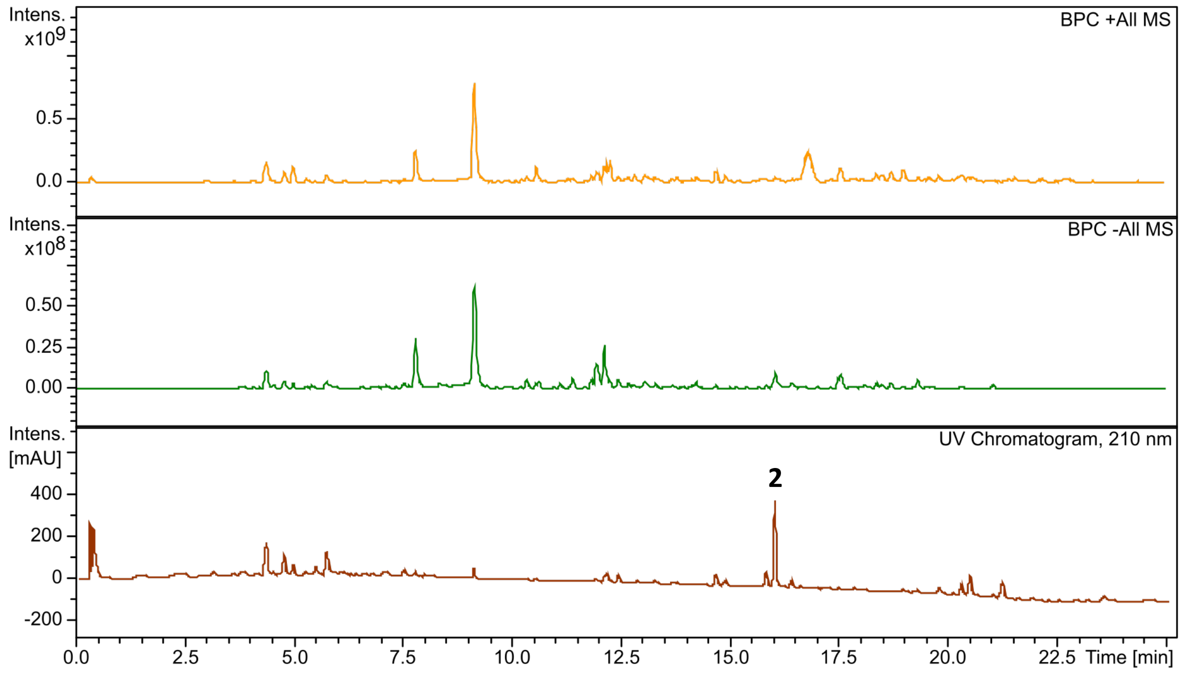

Figure S3: LC-ESI-MS spectra of methanol extracts of *P. frankenii* V16 (DSM 106521) cultivated on WOFT incubated for four week. Top-bottom: +MS, -MS, UV-chromatogramm at 210 nm. Numbers represents isolated talaroderxine C (**2**).

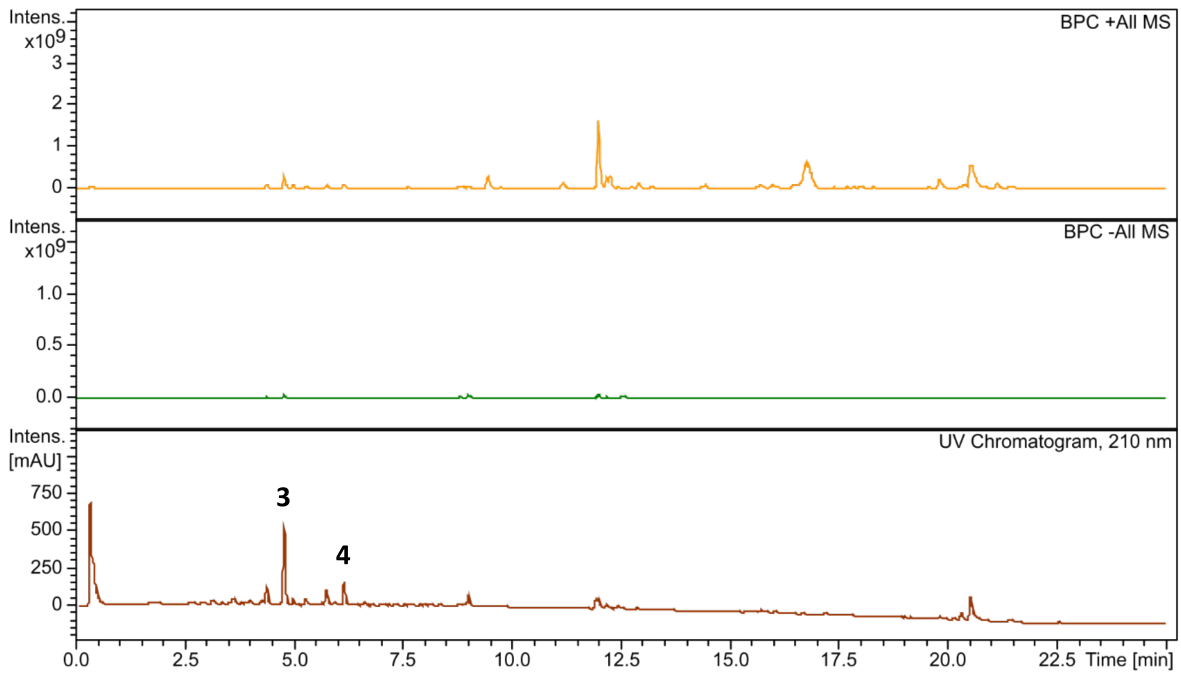

Figure S4: LC-ESI-MS spectra of mycelia extracts of *P. frankenii* V16 (DSM 106521) cultivated in YM6.3 mediaincubated for four week. Top-bottom: +MS, -MS, UV-chromatogramm at 210 nm. Numbers represents isolated outovirin B (**3**) and (3*S*,6*S*)-3,6-dibenzylpiperazine-2,5-dione **4**.

Table S1. 2D (COSY, HMBC and ROESY) NMR correlations of **1** in DMSO-d_6 at 500 MHz.

**Pos.**	**^{1}H-^{1}H COSY**[a]	**HMBC**[a]	**ROESY**[a]
1			
3	H_2-4, H_2-11		H_2-12
4	H-3, H-5w	3, 4a, 5w, 10a, 11	
4a			
5	H_2-4w	4, 5aw, 6, 9a, 10a	H_2-4, H-6
5a			
6	H-8		H-5
7			
8	H-6	6, 7, 9, 9a	
9			
9a			
10			
10a			
11	H-3, H_2-12	3, 4, 12, 13	
12	H_2-11, H_2-13	11, 13, 14	H-3
13	H_2-12, H_2-14	12, 14, 15	
14	H_2-13, H_3-15	13, 15	

[a] ”w” denotes weak correlation.

Table S2. 2D (COSY, HMBC and ROESY) NMR correlations of **2** in in DMSO-d_6 at 500 MHz.

**Pos.**	**^{1}H-^{1}H COSY**	**HMBC**	**ROESY**
1			
2	2-C$\underline{H}_3$	C-1, 2-$\underline{C}H_3$, C-4	H-5
3-NO*H*			
4			
5		C-1, C-4, C-7, C-8,	H-2
6-N*H*			
7		C-1w[a], C-4, C-5, C-8, C-9, C-16	H-15
8			
9	C$\underline{H}_2$-7w[a]	C-5w[a], C-7, C-8, C-11, C-16	
10-N*H*			
11			
12	H-13, H-14w[a]	C-14, C-16	
13	H-12, H-14, H-15w[a]	C-11, C-12w[a], C-14w[a], C-15	
14	H-12w[a], H-13, H-15	C-12, C-13w[a], C-15w[a], C-16	
15	H-13w[a], H-14, H-15	C-8, C-11, C-13, C-16w[a]	C$\underline{H}_2$-7
16			
2-Me	H-2	C-1, C-2	

[a] ”w” denotes weak correlation.

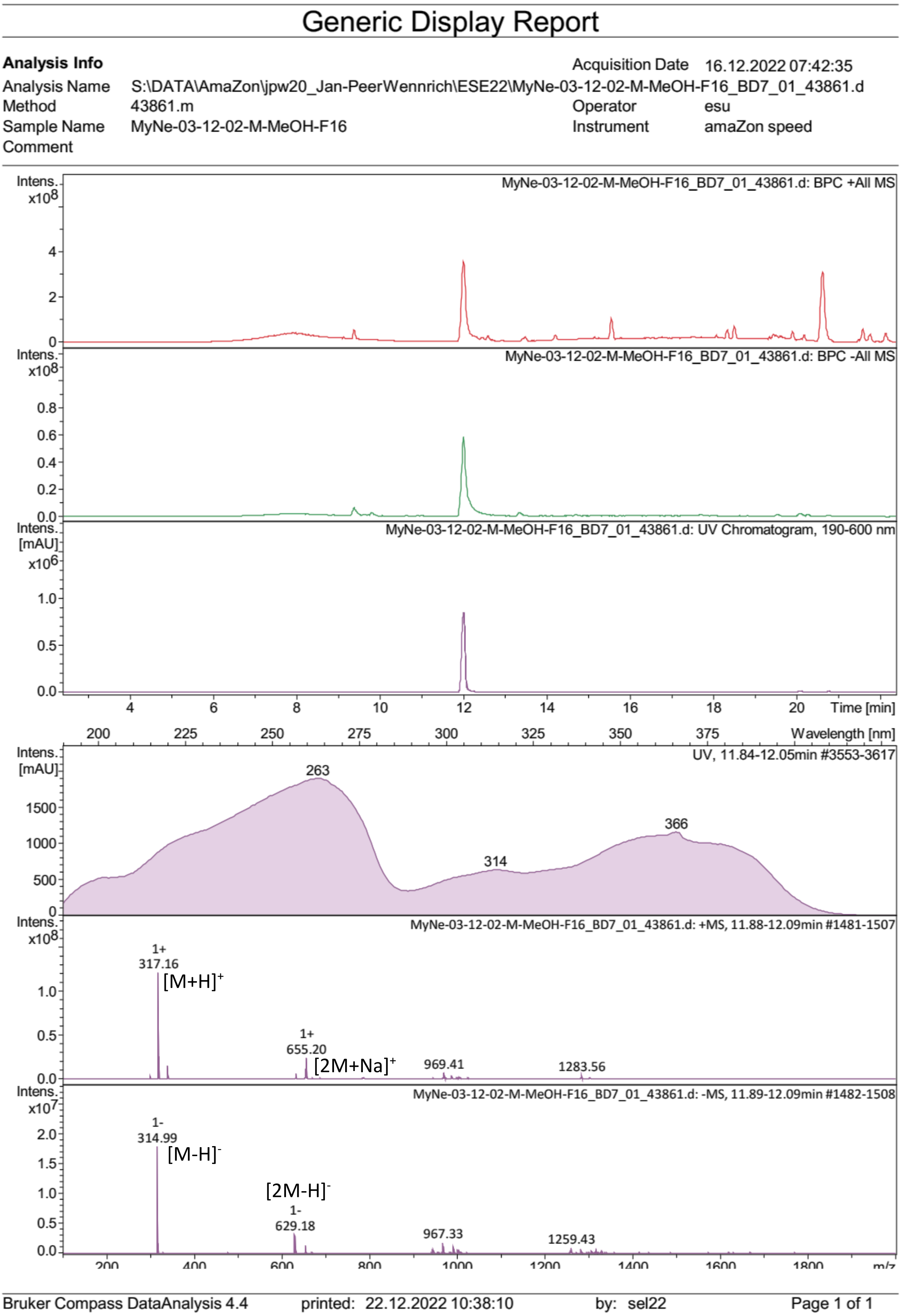

Figure S5. LRESIMS of **1**.

Generic Display Report

Analysis Info

		Acquisition Date	13.01.2023 12:41:11
Analysis Name	S:\DATA\MaXis\ESE22_Ellen Sepanian\23_01\MyNe-03-12-02-M-MeOH-F16_23_01_11193.d		
Method	pos_säure_10000_screening_ms_100_2500_line.m	Operator	ate06
Sample Name	MyNe-03-12-02-M-MeOH-F16	Instrument	maXis
Comment	Screening01<br>Waters Acquity UPLC BEH C_18 1,7um 2.1x50mm		

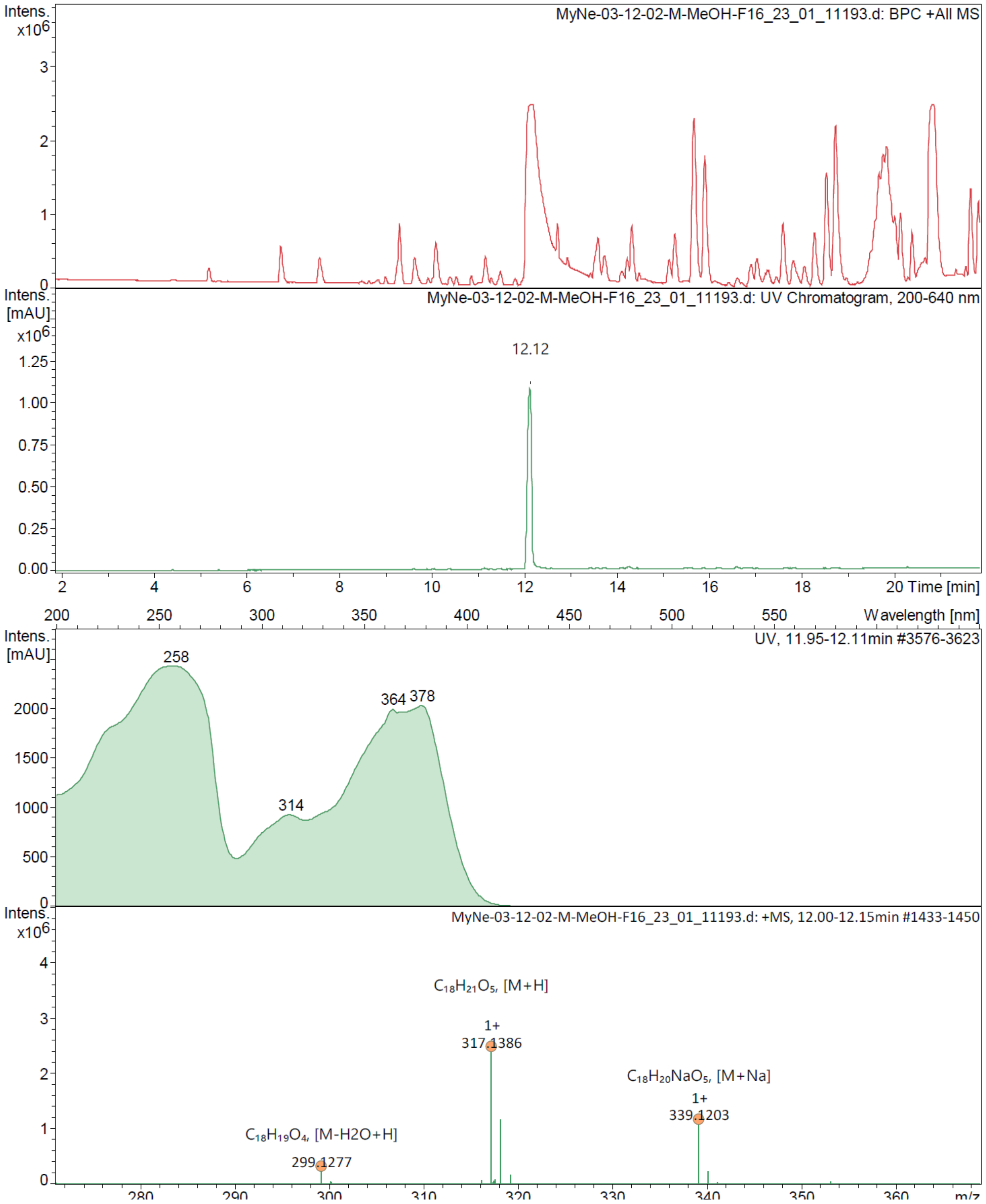

Bruker Compass DataAnalysis 4.4 printed: 07.06.2023 19:05:42 by: sel22 Page 1 of 1

Figure S6. HRESIMS of **1**.

Data Set: F16 - RawData

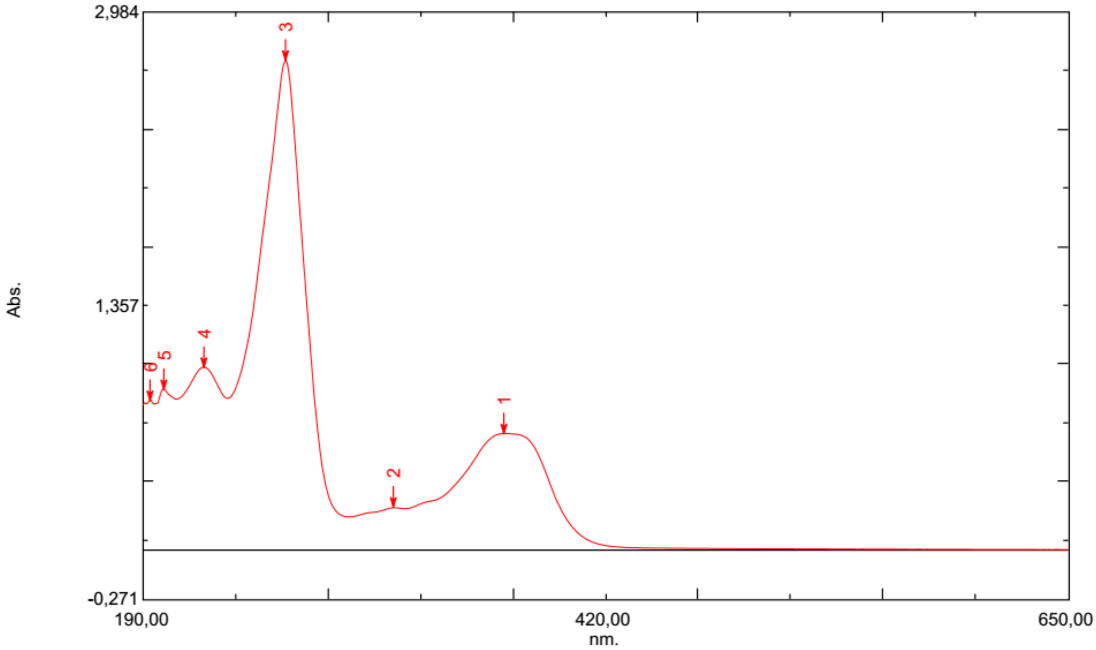

Measurement Properties

Wavelength Range (nm.): 190,00 to 650,00
Scan Speed: Medium
Sampling Interval: 0,5
Auto Sampling Interval: Disabled
Scan Mode: Single

Instrument Properties

Instrument Type: UV-2400PC Series
Measuring Mode: Absorbance
Slit Width: 2,0 nm
Light Source Change Wavelength: 360,0 nm
S/R Exchange: Normal

[illegible]

No.	P/V	Wavelength	Abs.	Description
1	↑	369,50	0,644	
2	↑	314,50	0,232	
3	↑	261,00	2,713	
4	↑	220,50	1,013	
5	↑	200,00	0,890	
6	↑	193,50	0,830	
7	↓	320,00	0,227	
8	↓	292,50	0,181	
9	↓	232,00	0,840	
10	↓	206,50	0,836	
11	↓	196,00	0,808	

Figure S7. UV spectrum of **1**.

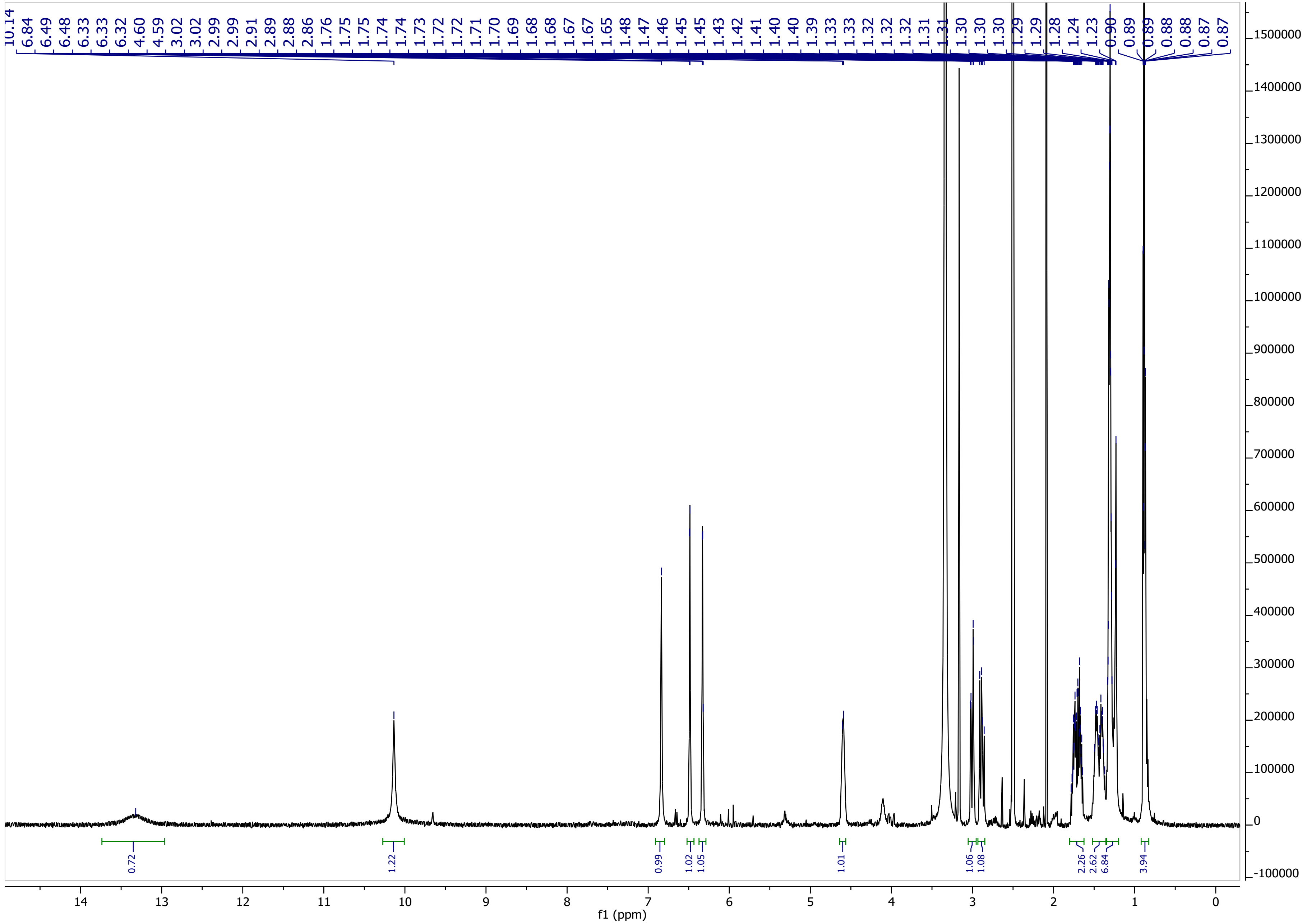

Figure S8. ^{1}H NMR spectrum of **1** in DMSO-d_6 at 500 MHz.

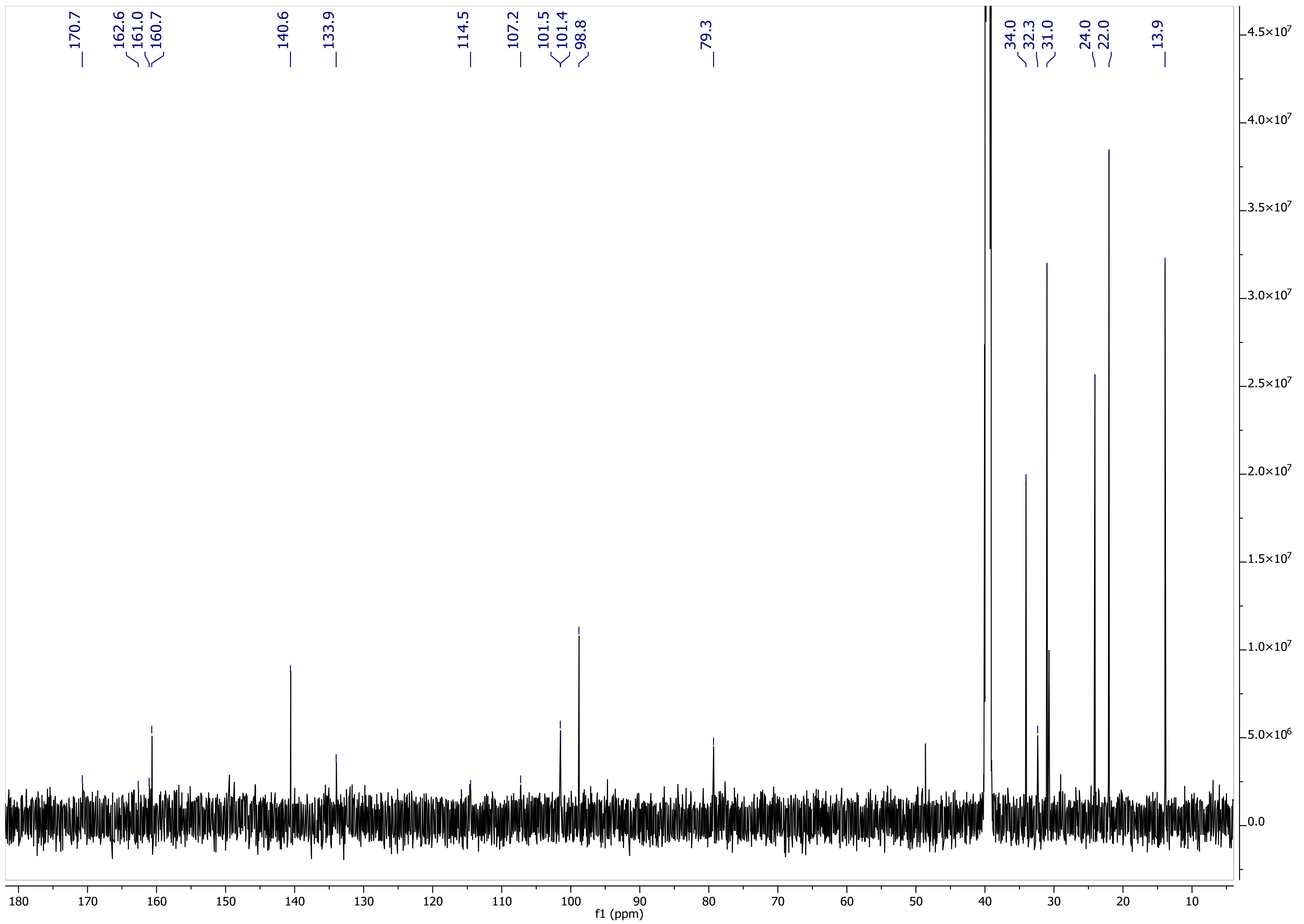

Figure S9. ^{13}C NMR spectrum of **1** in DMSO-d_6 at 125 MHz.

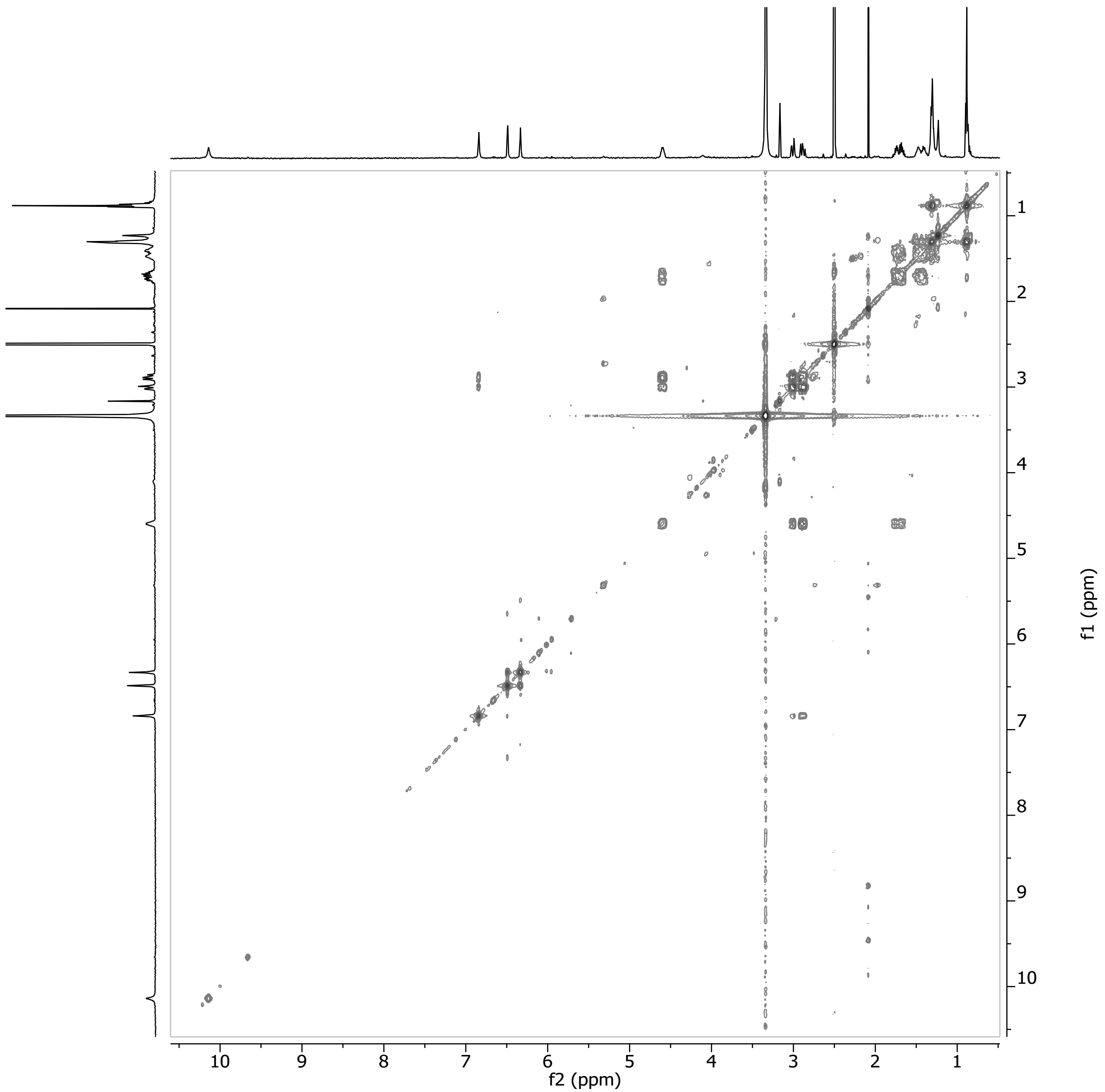

Figure S10. ^{1}H-^{1}H COSY spectrum of **1** in DMSO-d_6 at 500 MHz.

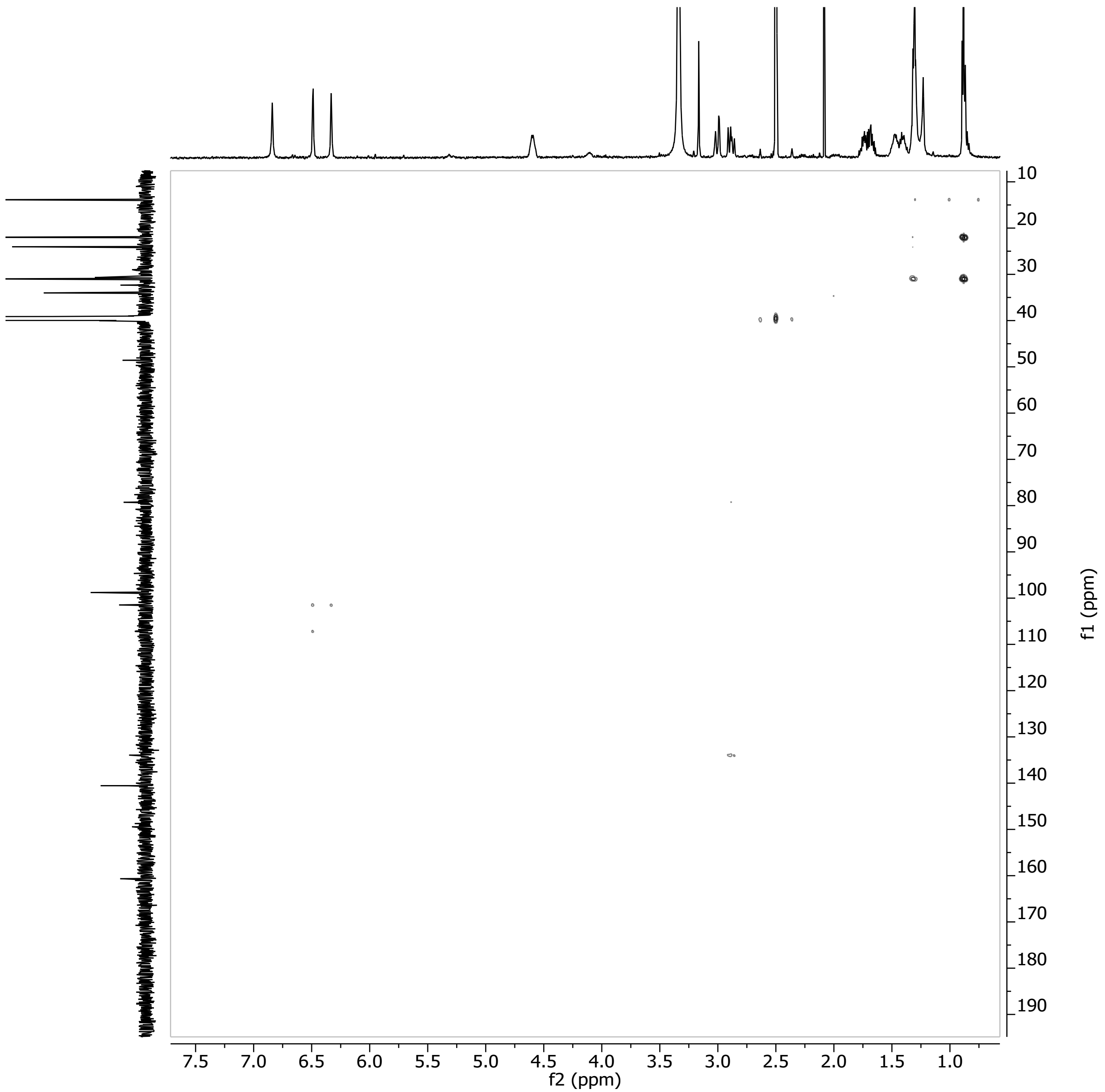

Figure S11. HMBC spectrum of **1** in DMSO-d_6 at 500 MHz.

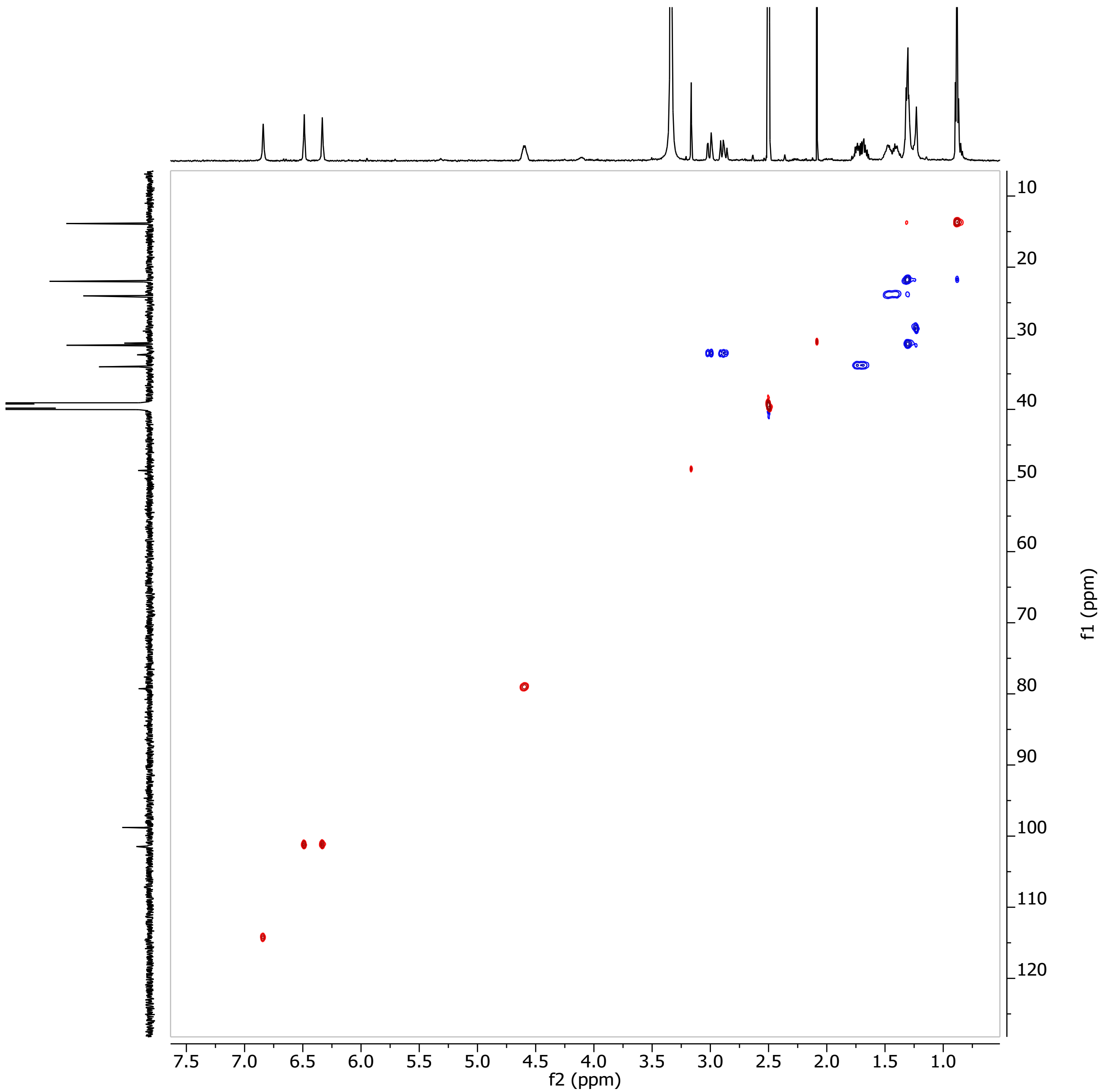

Figure S12. HSQC spectrum of **1** in DMSO-d_6 at 500 MHz.

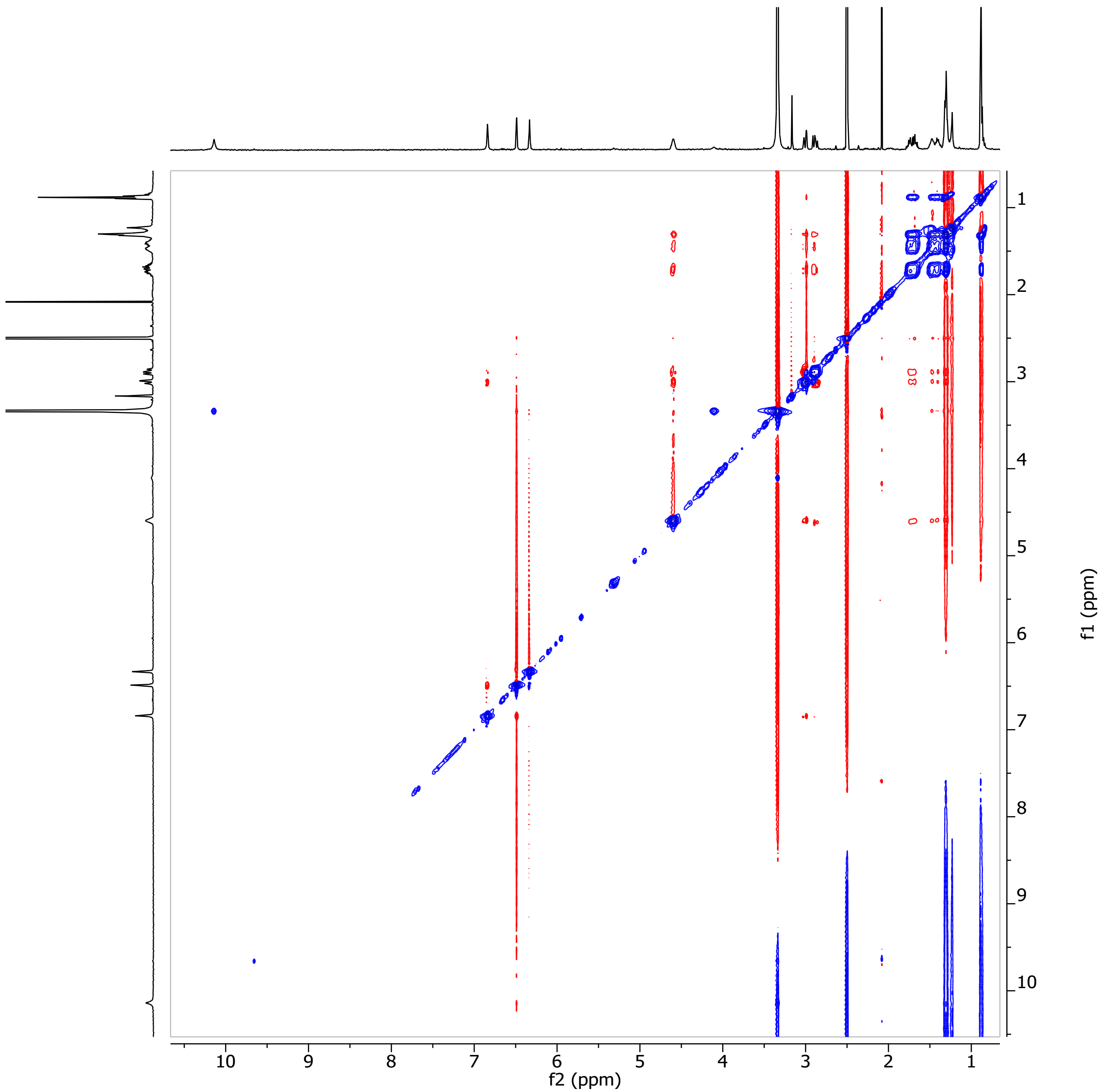

Figure S13. ROESY spectrum of **1** in DMSO-d_6 at 500 MHz.

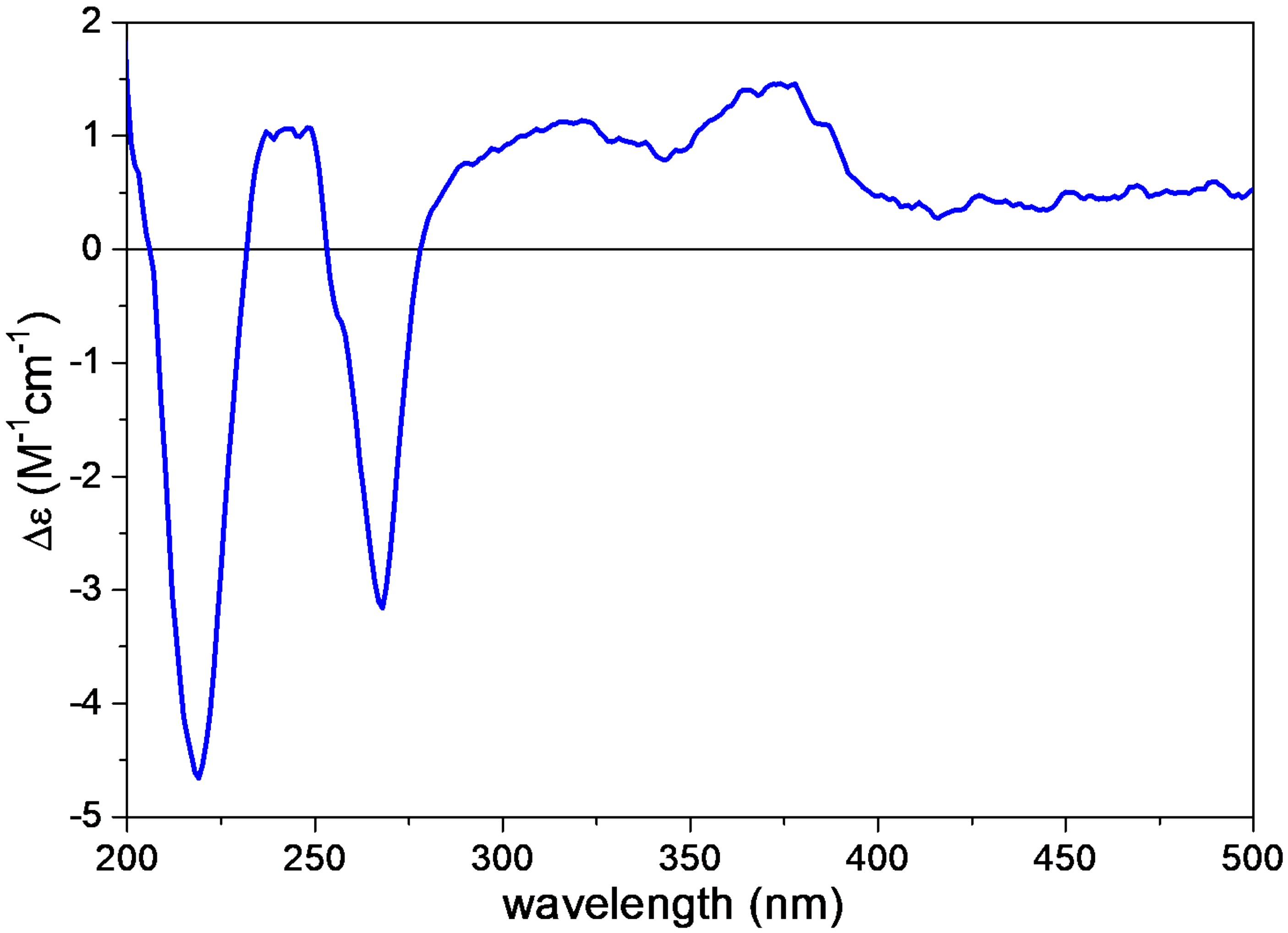

Figure S14. Experimental ECD spectrum of semitalaroderxine C (**1**) in methanol.

Generic Display Report

Analysis Info

Analysis Name S:\DATA\AmaZon\jpw20_Jan-Peer Wennrich\MycoNem_HPLC\MyNe_11\01-11-06+07-MeOH-F7-F10\MyNe-11-06+07-MeOH-F7-F10-F3_GB3_01_43449.d
Method 43449.d
Sample Name MyNe-11-06+07-MeOH-F7-F10-F3
Comment

Acquisition Date 30.11.2022 17:22:46
Operator esu
Instrument amaZon speed

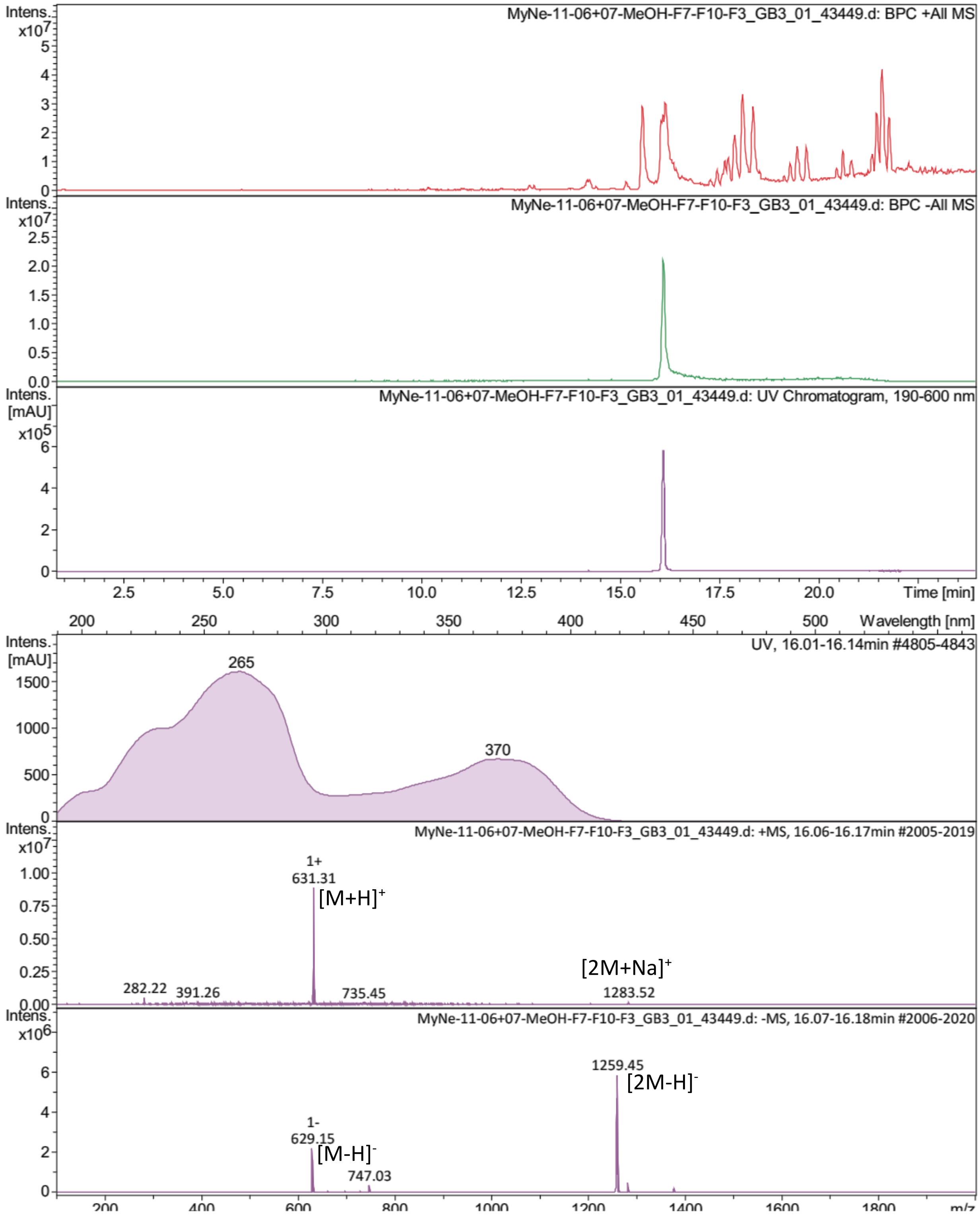

Figure S15. LRESIMS of **2**.

Generic Display Report

Analysis Info

Analysis Name	S:\DATA\MaXis\ESE22_Ellen Sepanian\22_12\MyNe-01-11-06+07-MeOH-F7-F10-F3_23_01_11174.d	Acquisition Date	13.12.2022 10:28:50
Method	pos_säure_10000_screening_ms_100_2500_line.m	Operator	ate06
Sample Name	MyNe-01-11-06+07-MeOH-F7-F10-F	Instrument	maXis
Comment	Screening01<br>Waters Acquity UPLC BEH C_18 1,7um 2.1x50mm		

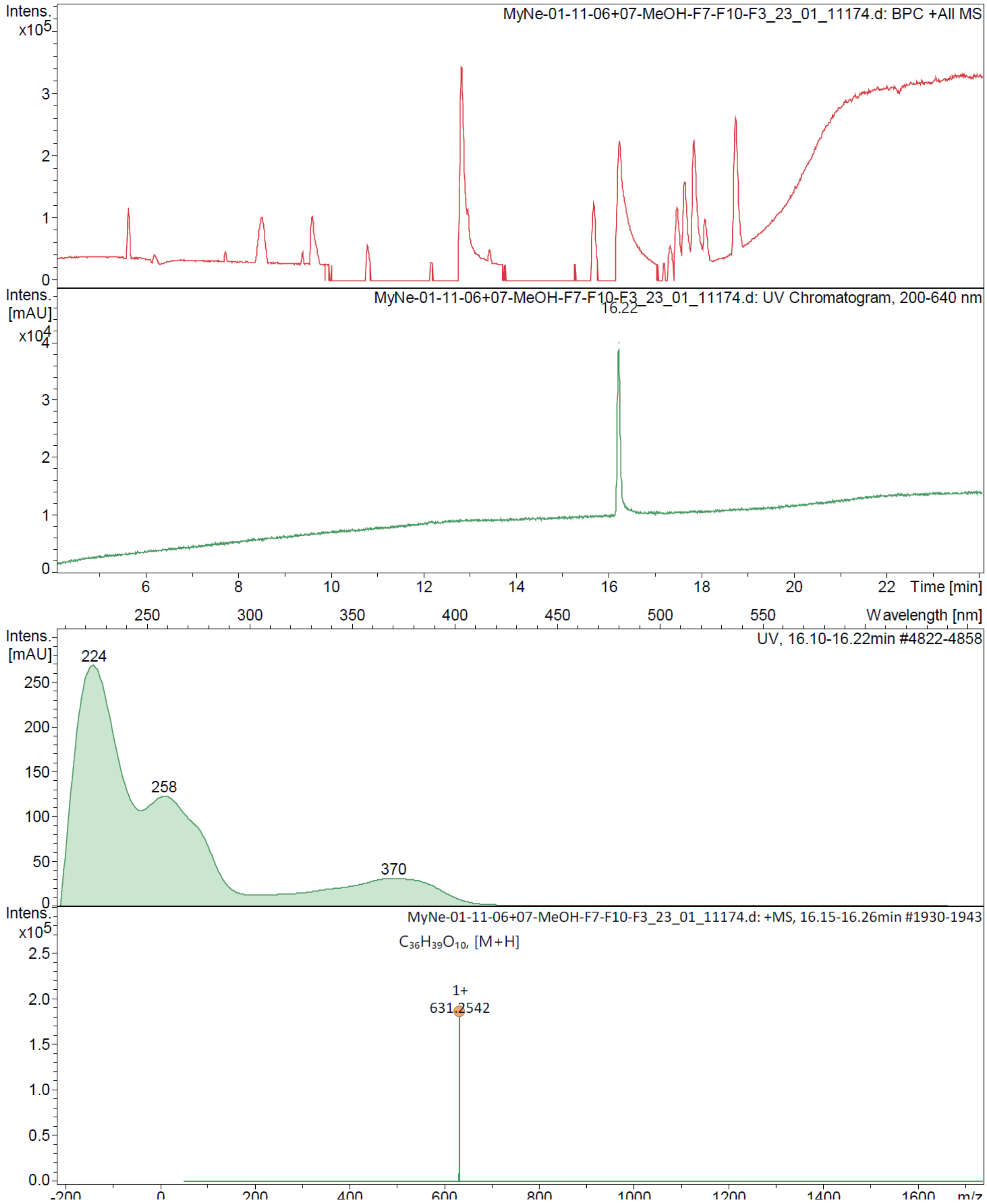

Figure S16. HRESIMS of **2**.

Data Set: F5_F15 - RawData

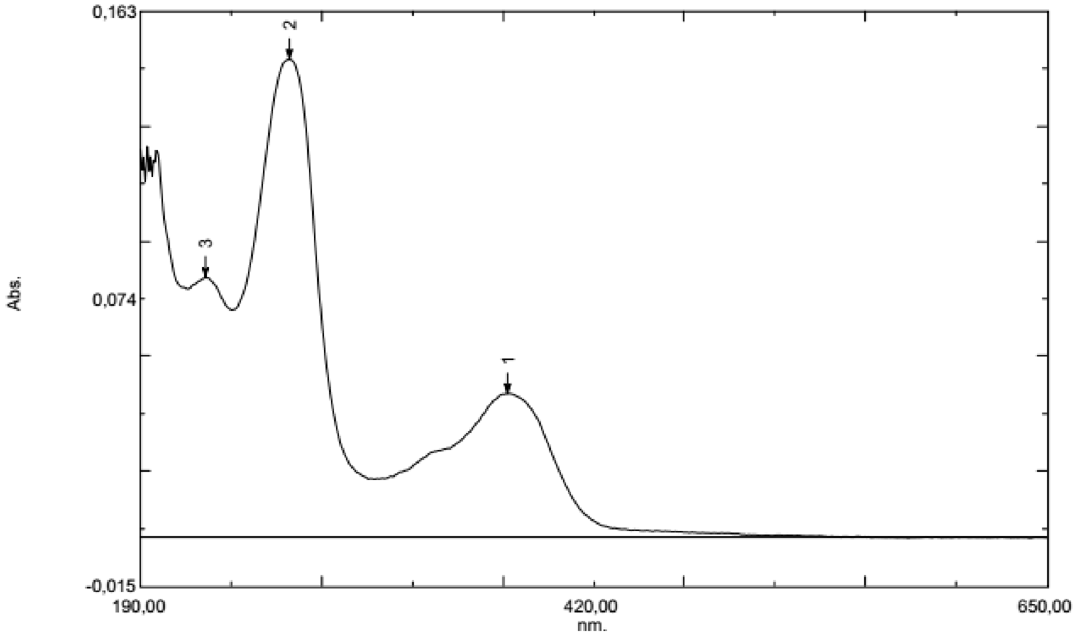

Measurement Properties

Wavelength Range (nm.):	190,00 to 650,00
Scan Speed:	Medium
Sampling Interval:	0,5
Auto Sampling Interval:	Disabled
Scan Mode:	Single

Instrument Properties

Instrument Type:	UV-2400PC Series

No.	P/V	Wavelength	Abs.	Description
1	↑	376,50	0,045	
2	↑	265,50	0,148	
3	↑	223,00	0,081	
4	↓	305,50	0,018	
5	↓	236,50	0,071	
6	↓	214,50	0,077	

Figure S17. UV spectrum of **2**.

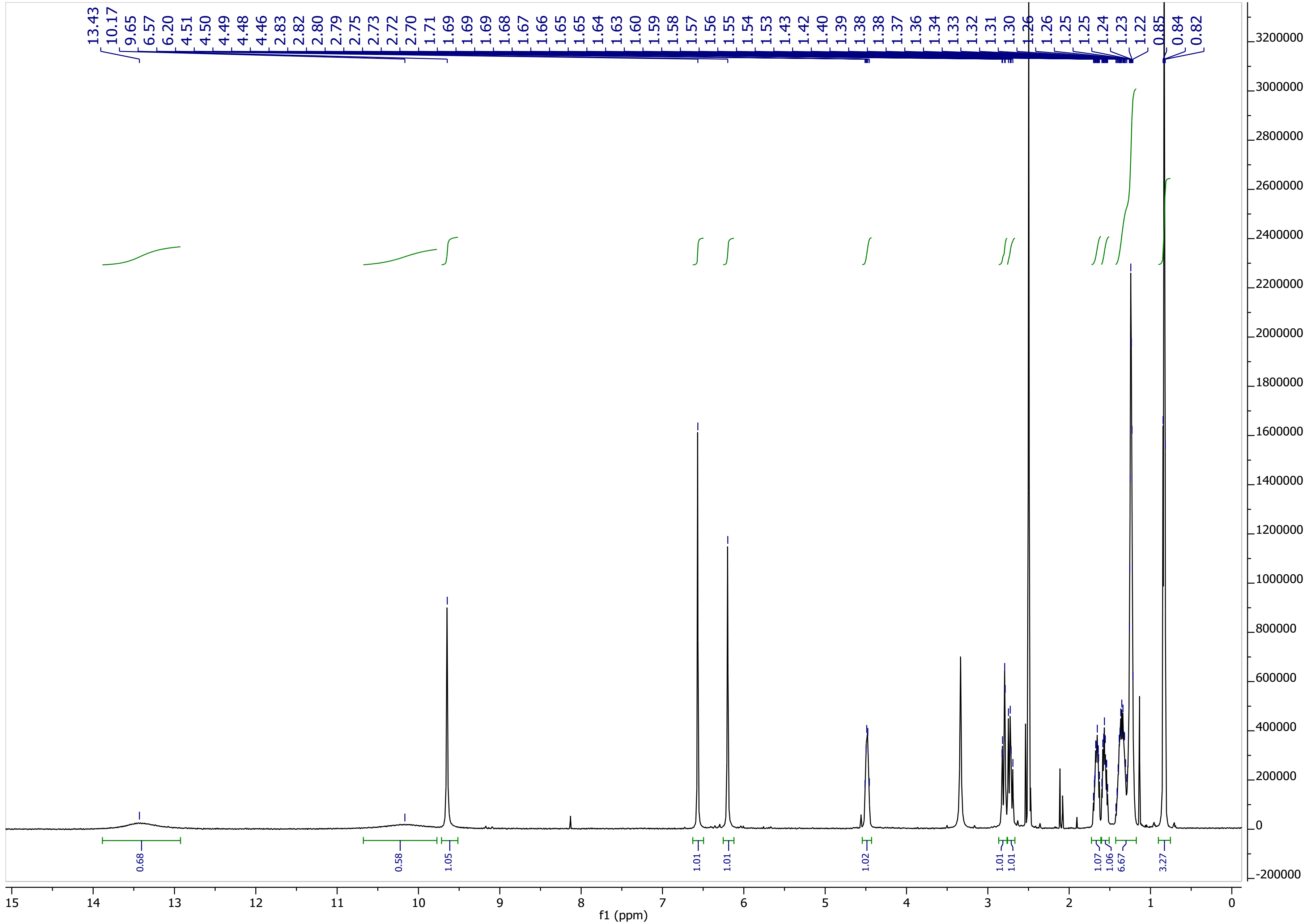

Figure S18. ^{1}H NMR spectrum of **2** in DMSO-d_6 at 500 MHz.

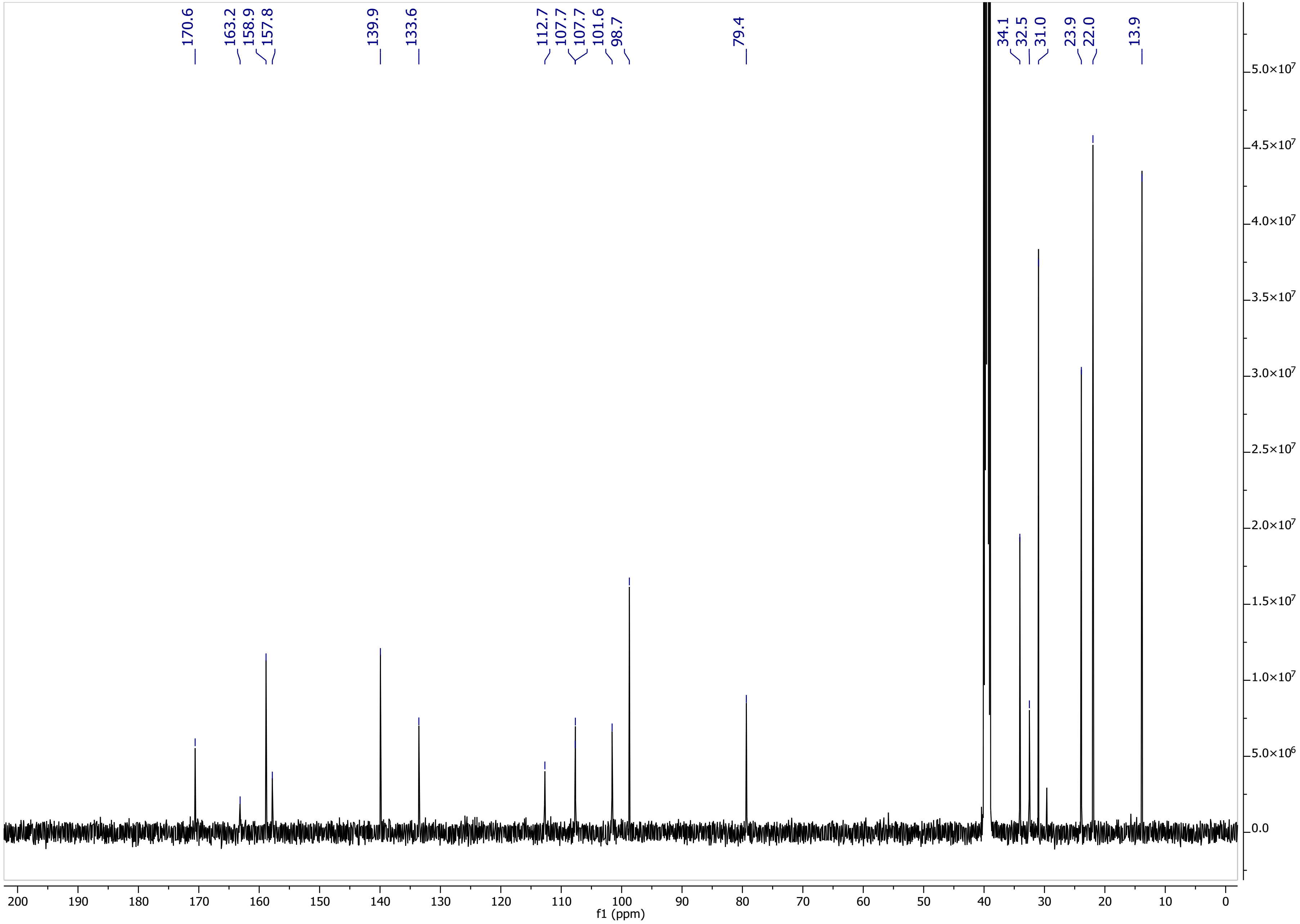

Figure S19. ^{13}C NMR spectrum of **2** in DMSO-d_6 at 125 MHz.

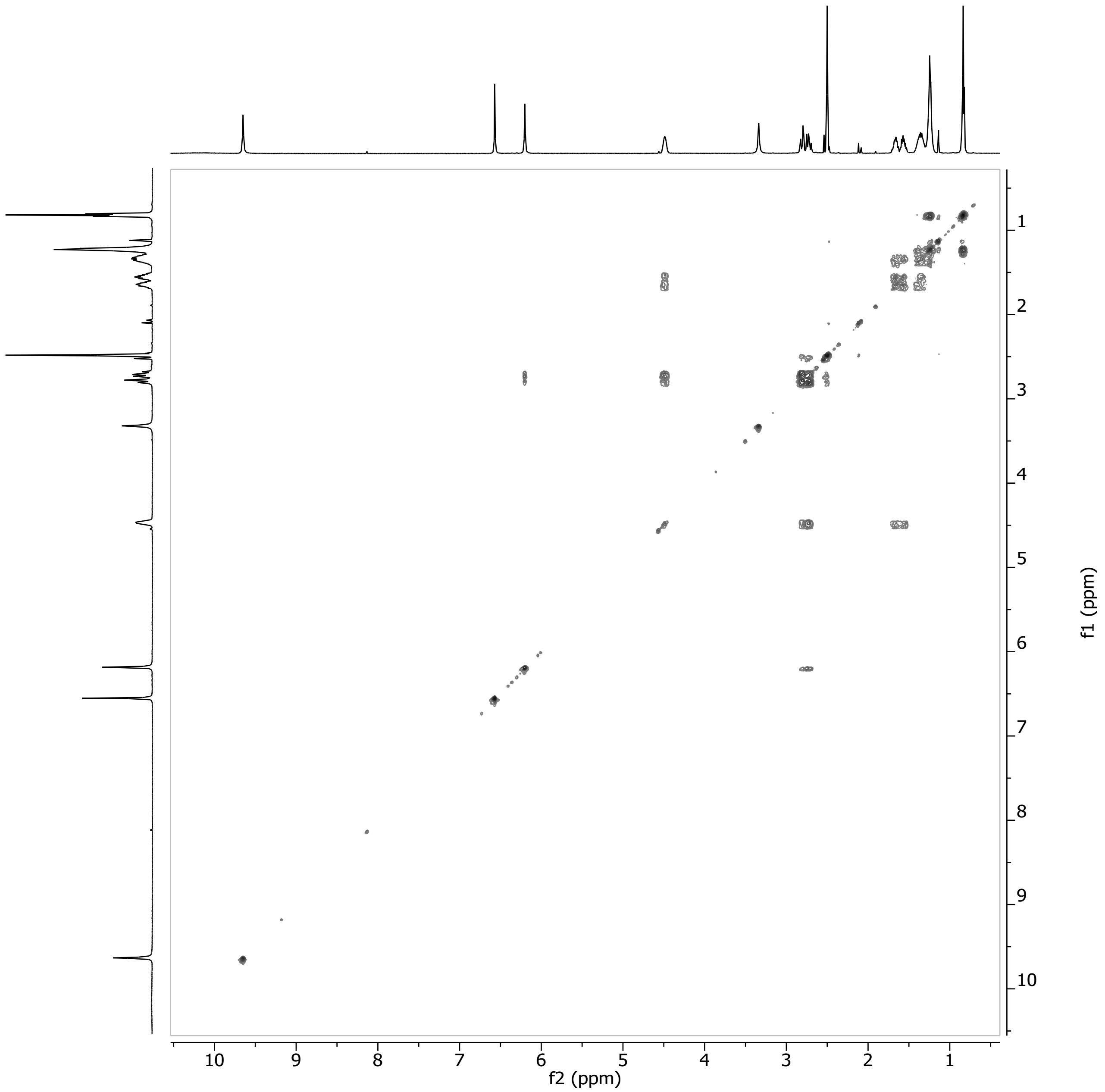

Figure S20. ^{1}H-^{1}H COSY spectrum of **2** in DMSO-d_6 at 500 MHz.

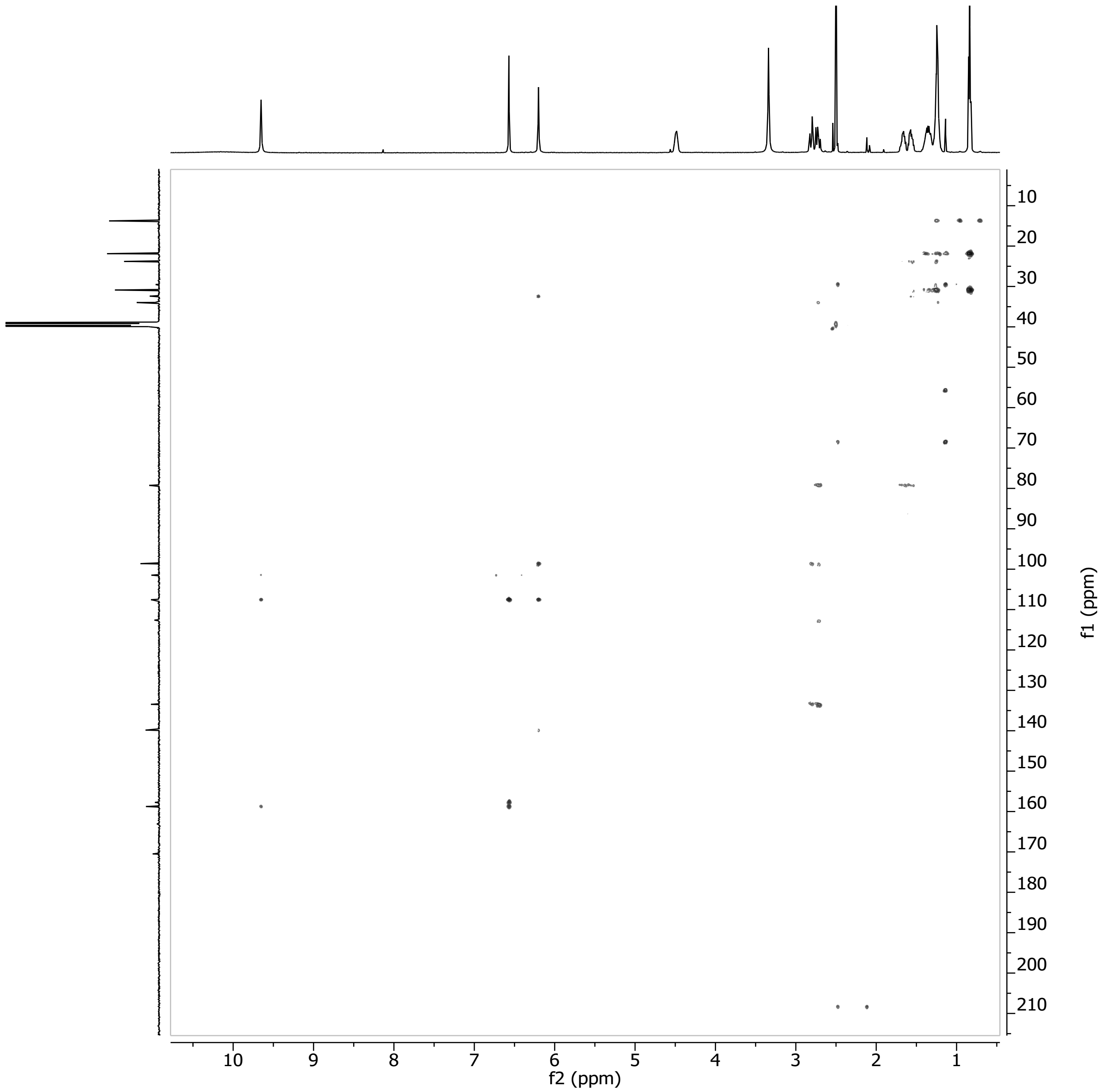

Figure S21. HMBC spectrum of **2** in DMSO-d_6 at 500 MHz.

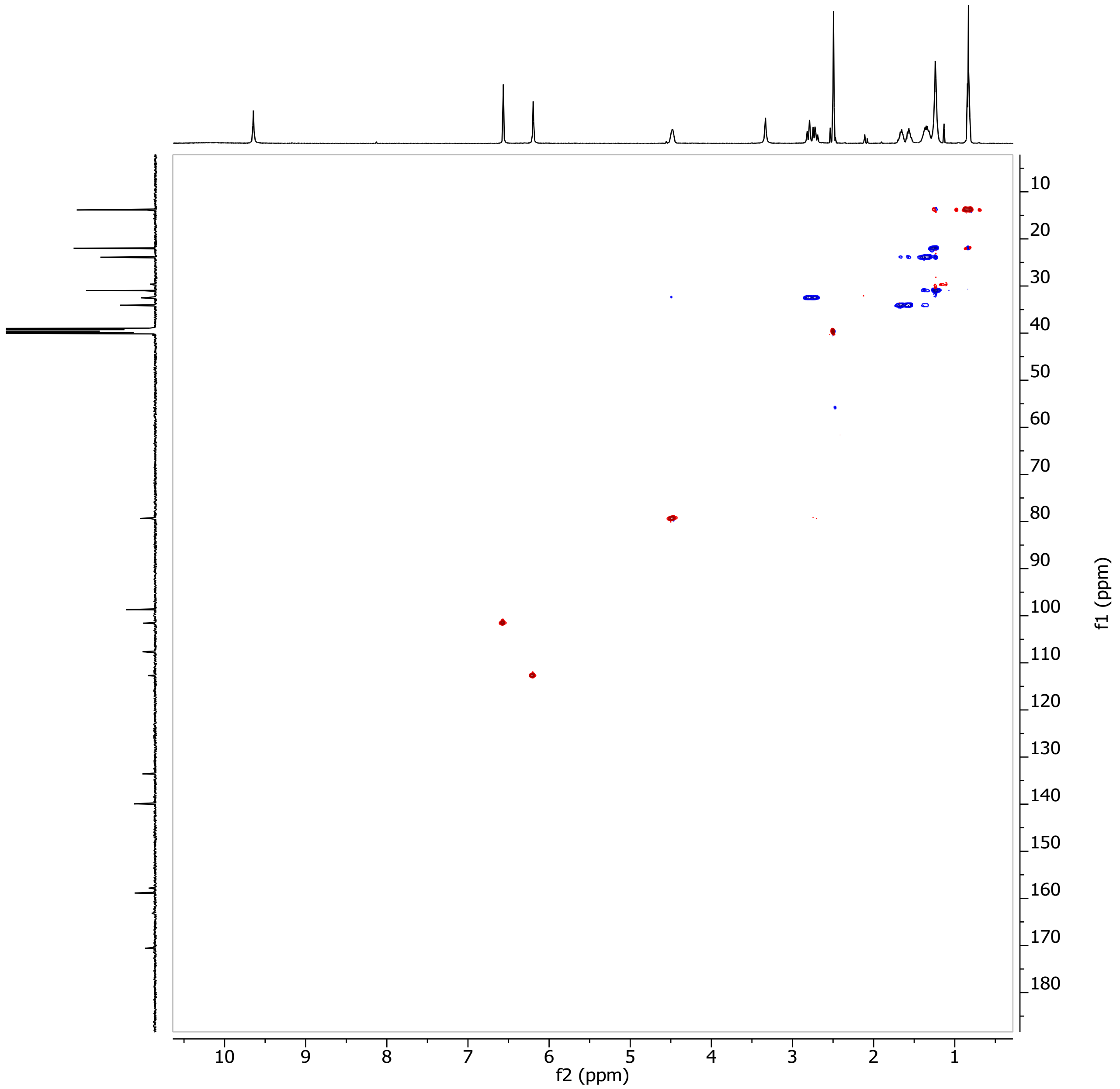

Figure S22. HSQC spectrum of **2** in DMSO-d_6 at 500 MHz.

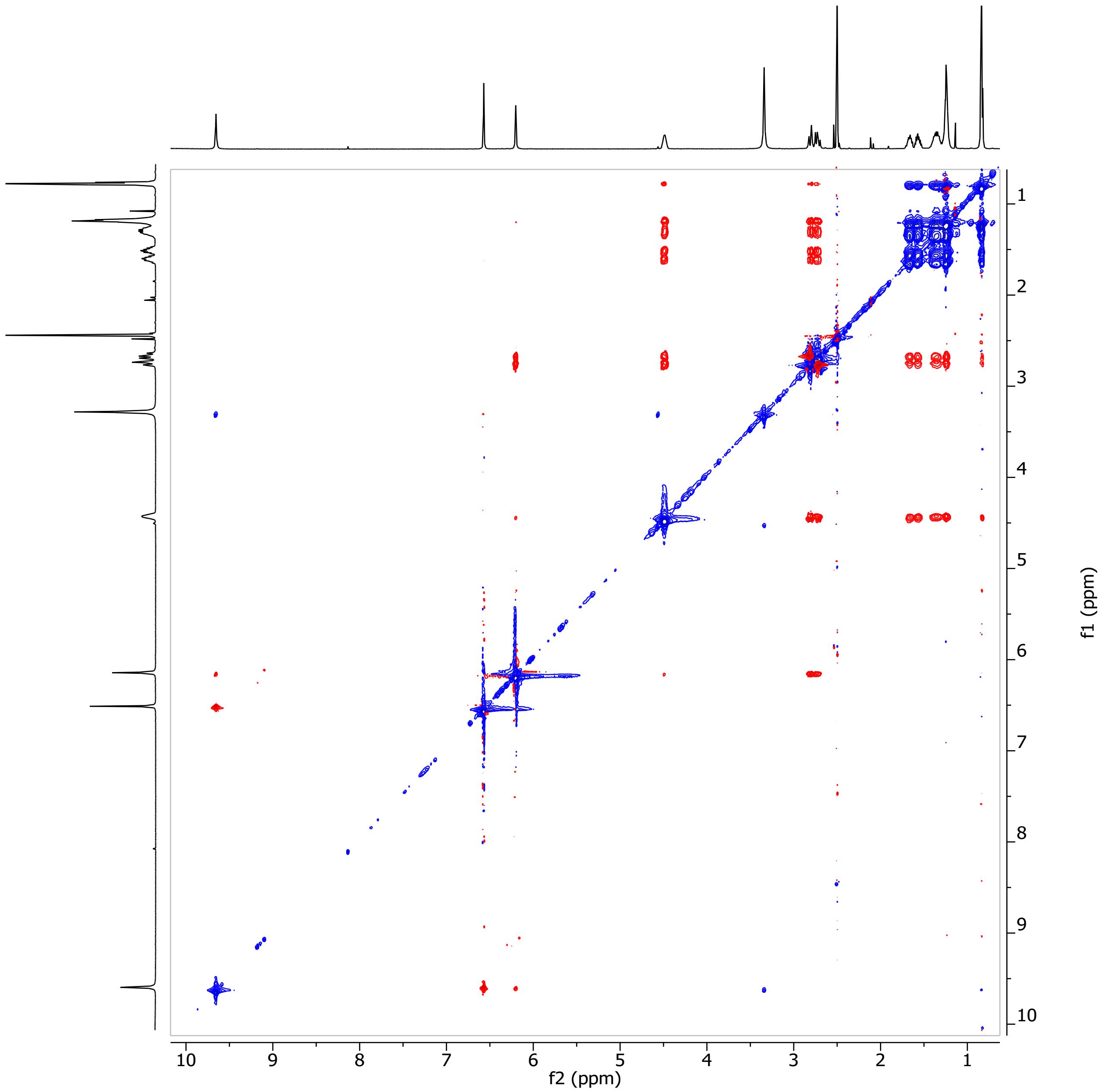

Figure S23. ROESY spectrum of **2** in DMSO-d_6 at 500 MHz.

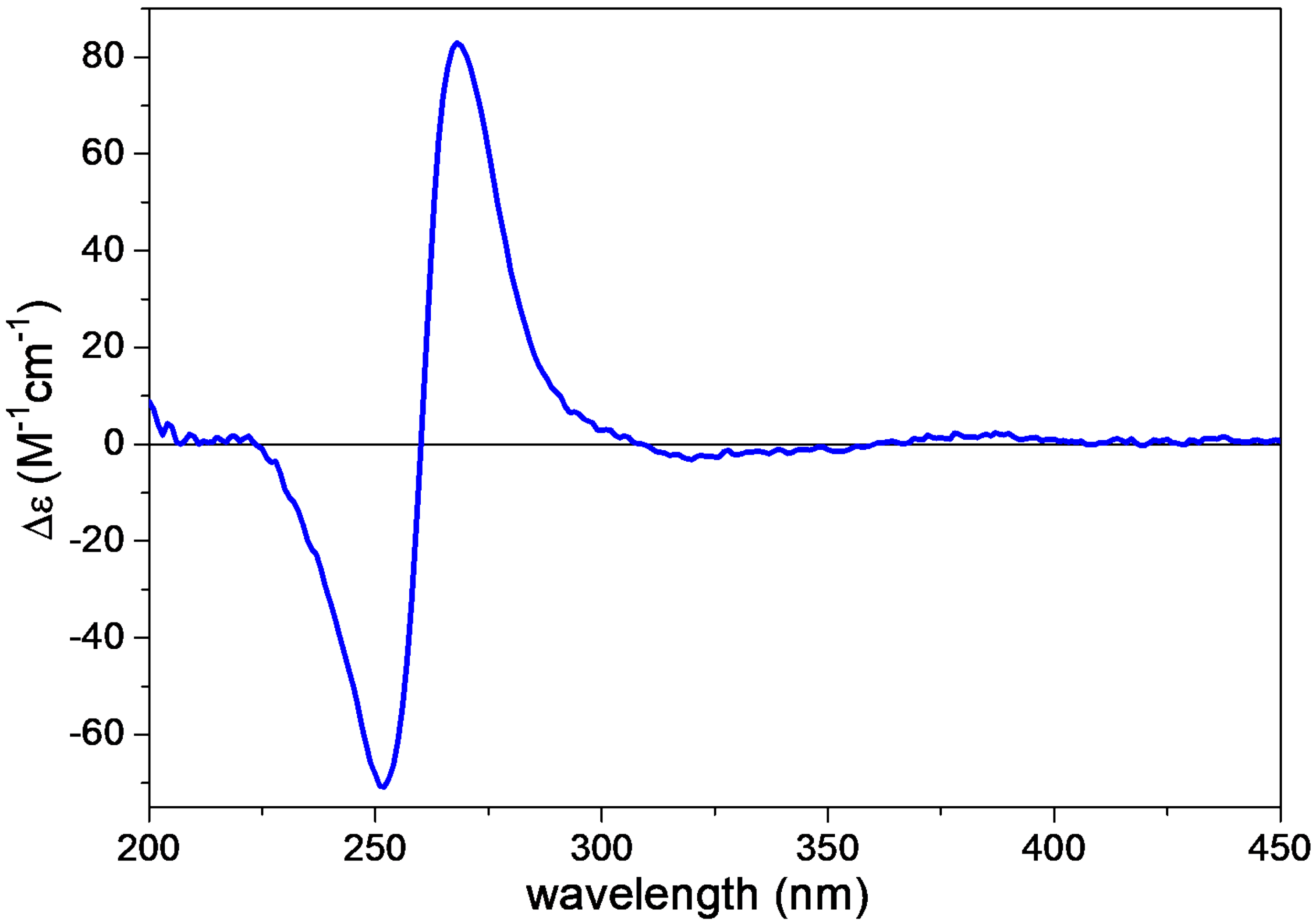

Figure S24. Experimental ECD spectrum of talaroderxine C (**2**) in methanol.

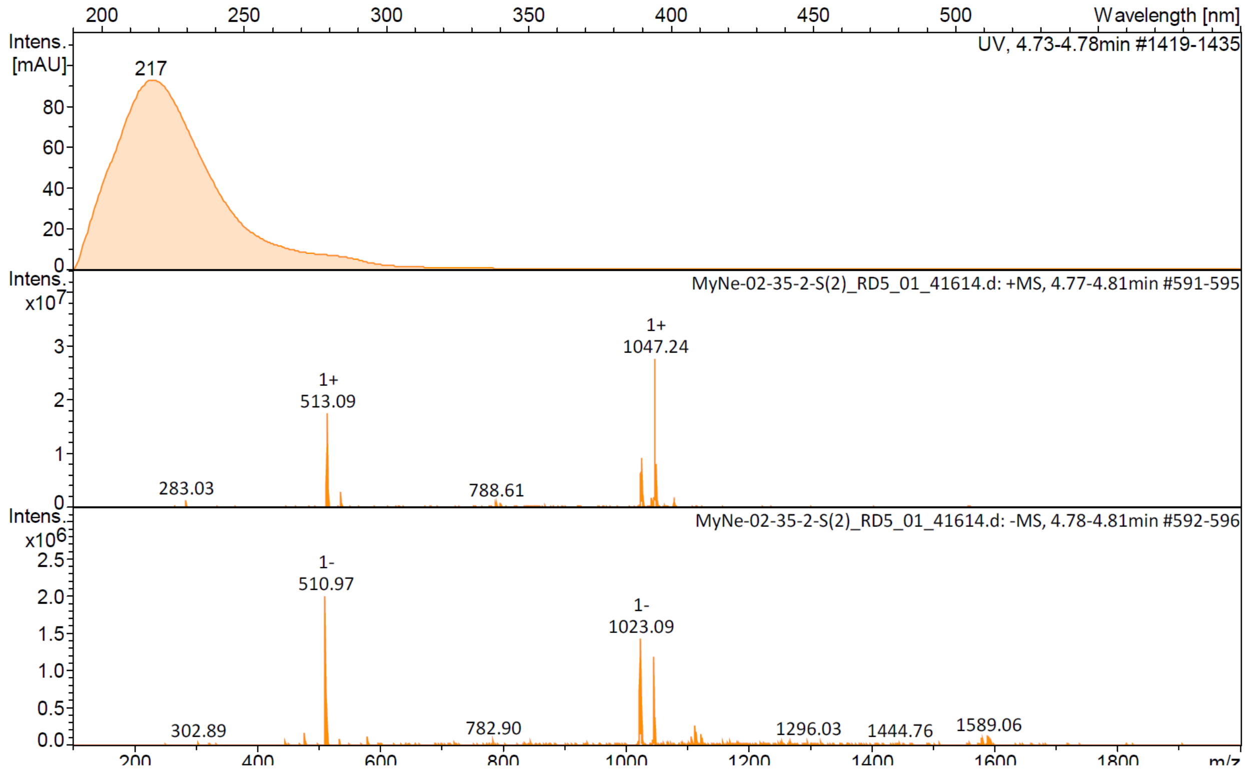

Figure S25. LRESIMS of **3**.

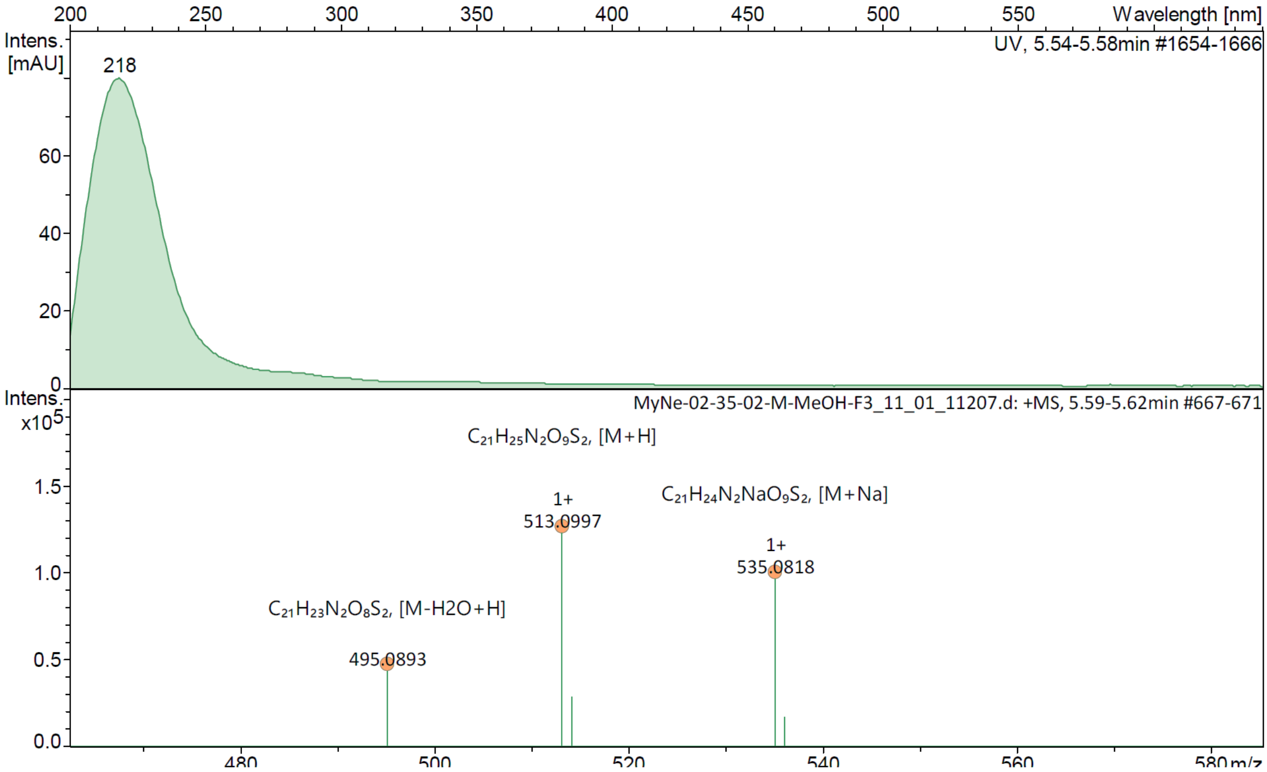

Figure S26. HRESIMS of **3**.

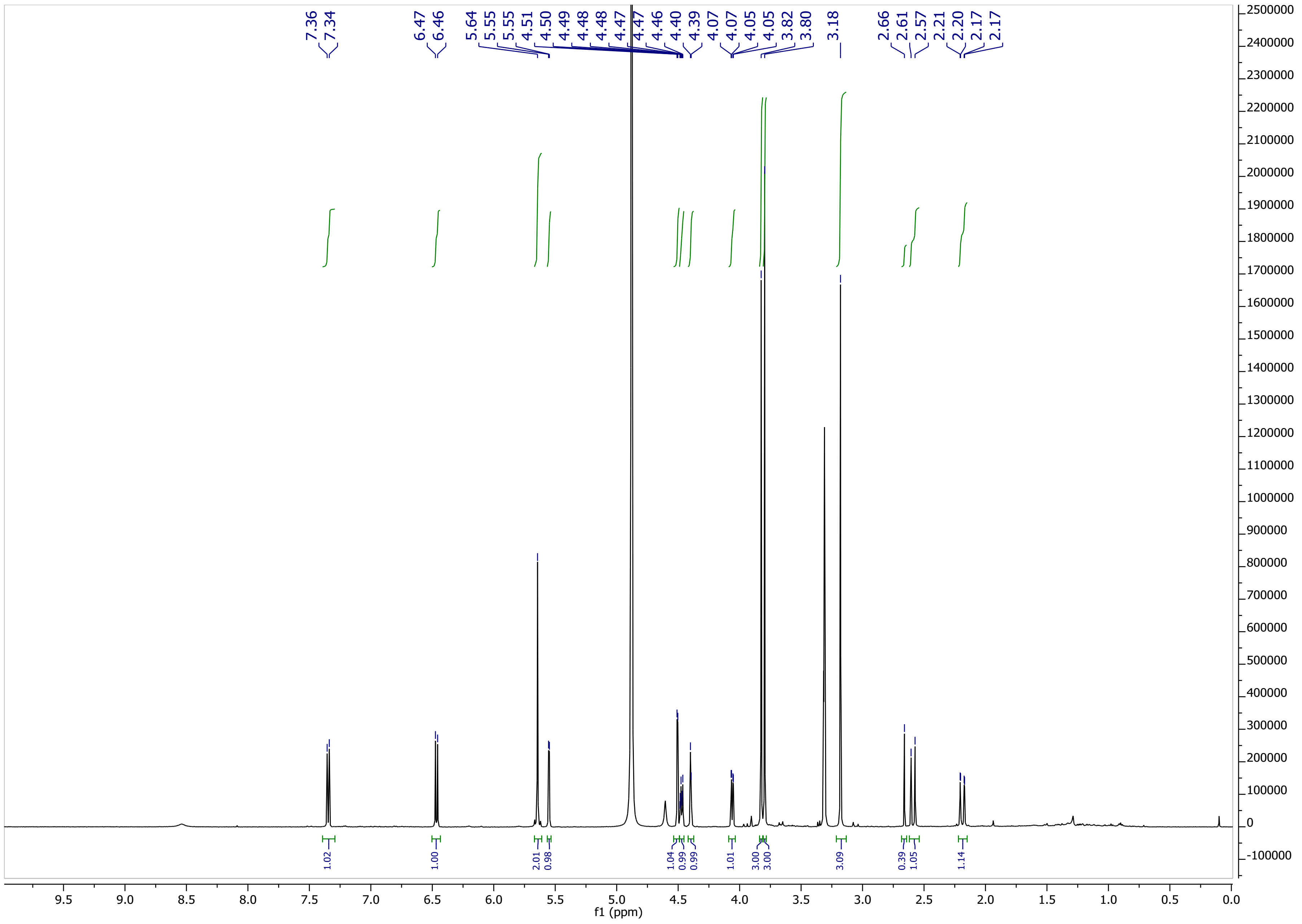

Figure S27. ^{1}H NMR spectrum of **3** in methanol-d_4 at 500 MHz.

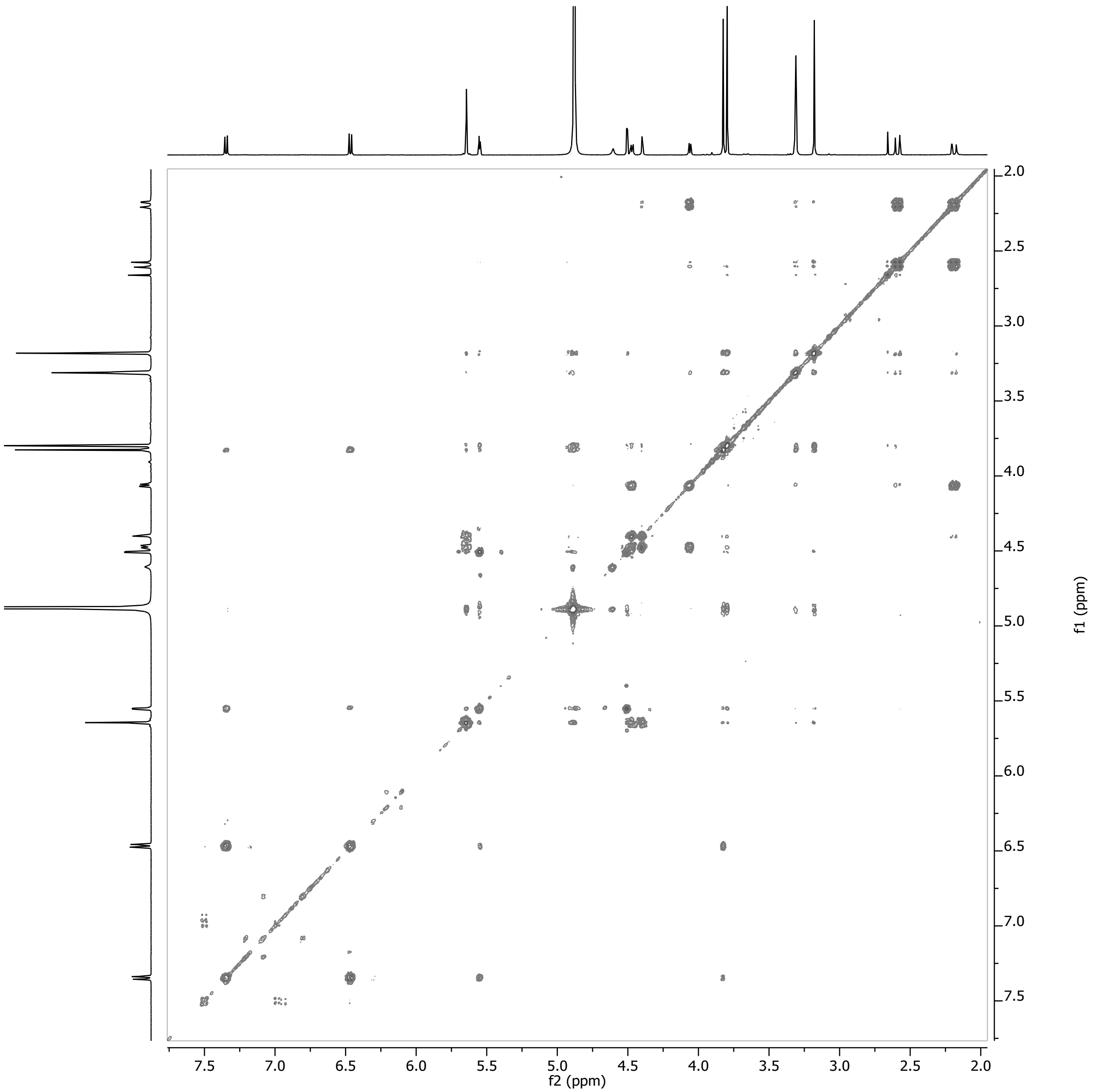

Figure S28. ^{1}H-^{1}H COSY spectrum of **3** in methanol-d_4 at 500 MHz.

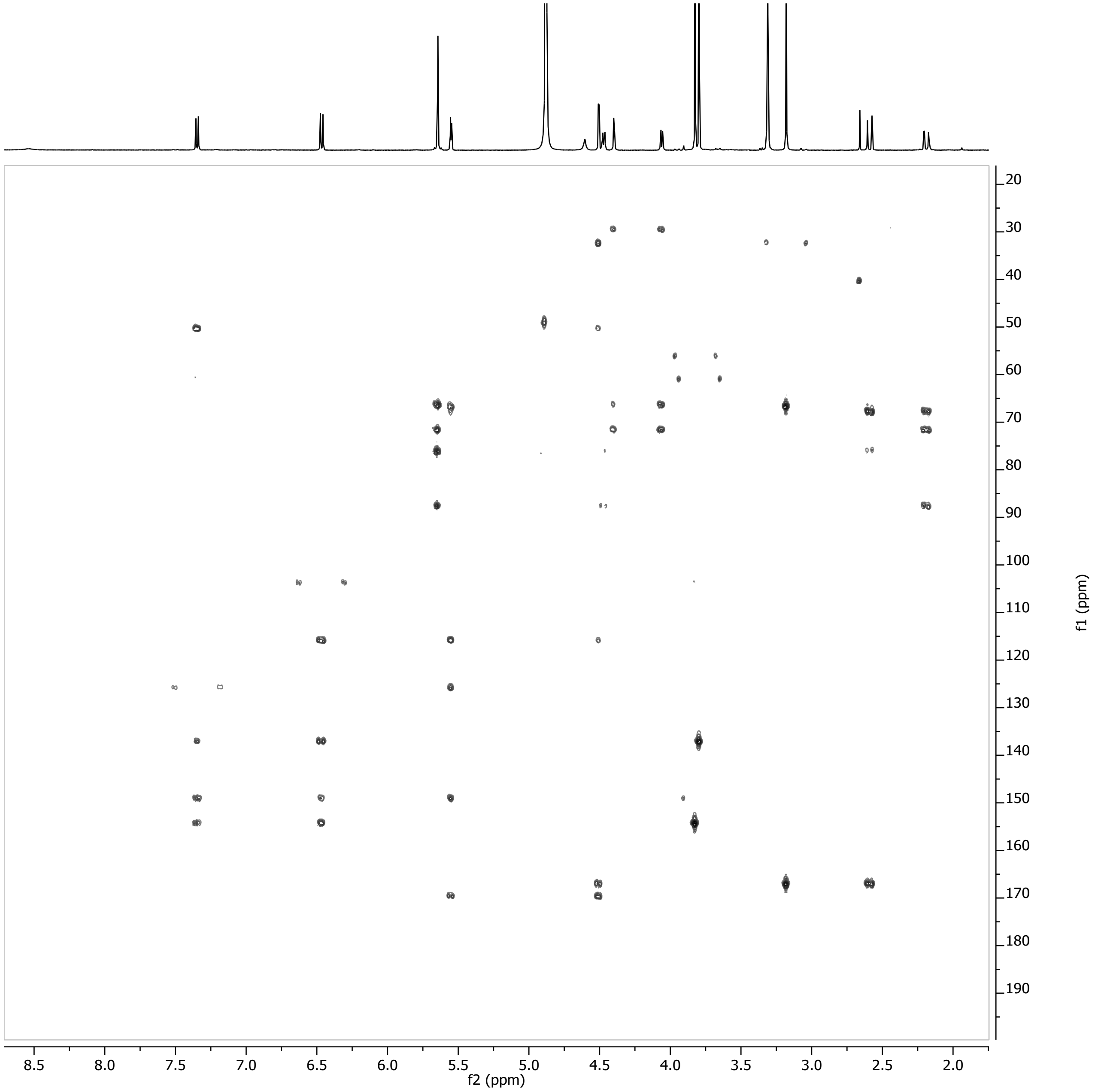

Figure S29. HMBC spectrum of **3** in methanol-d_4 at 500 MHz.

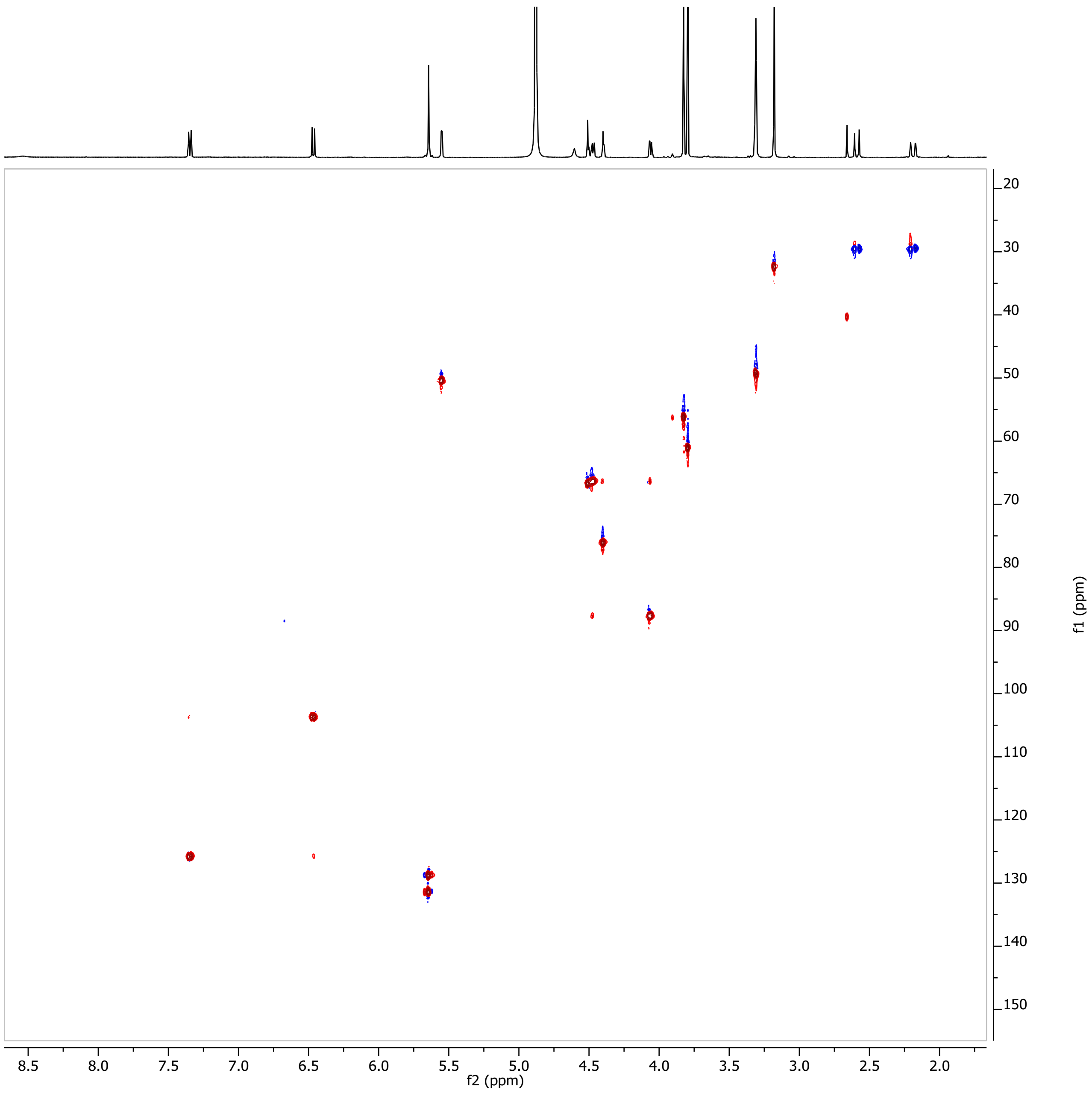

Figure S30. HSQC spectrum of **3** in methanol-d_4 at 500 MHz.

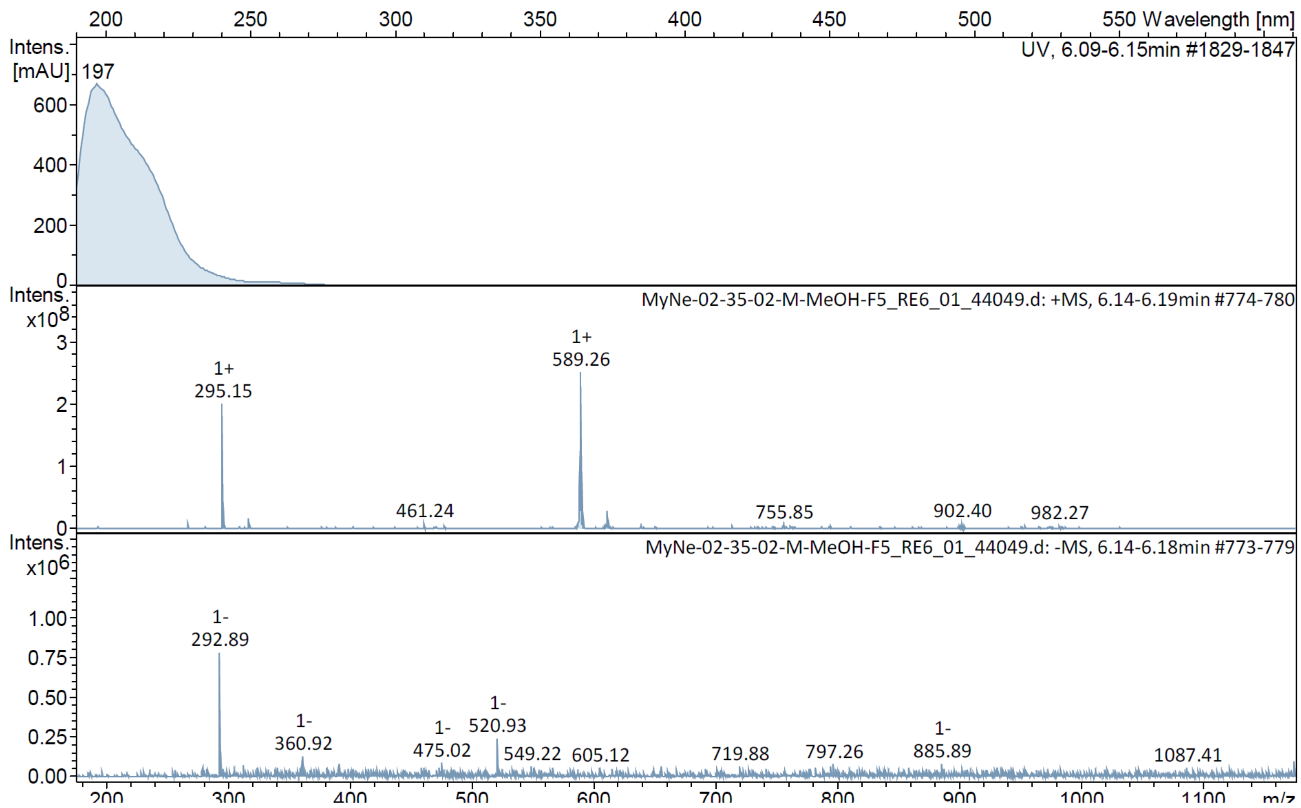

Figure S31. LRESIMS of **4**.

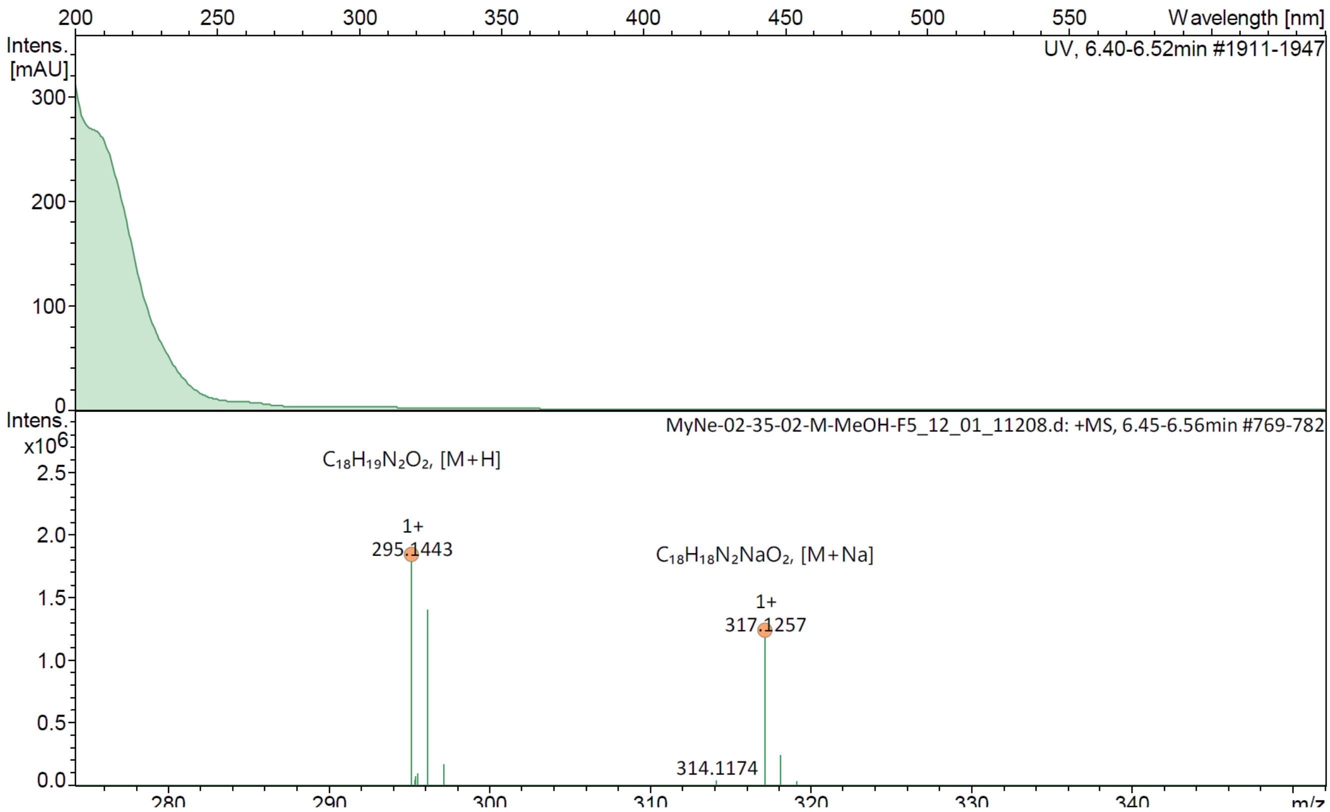

Figure S32. HRESIMS of **4**.

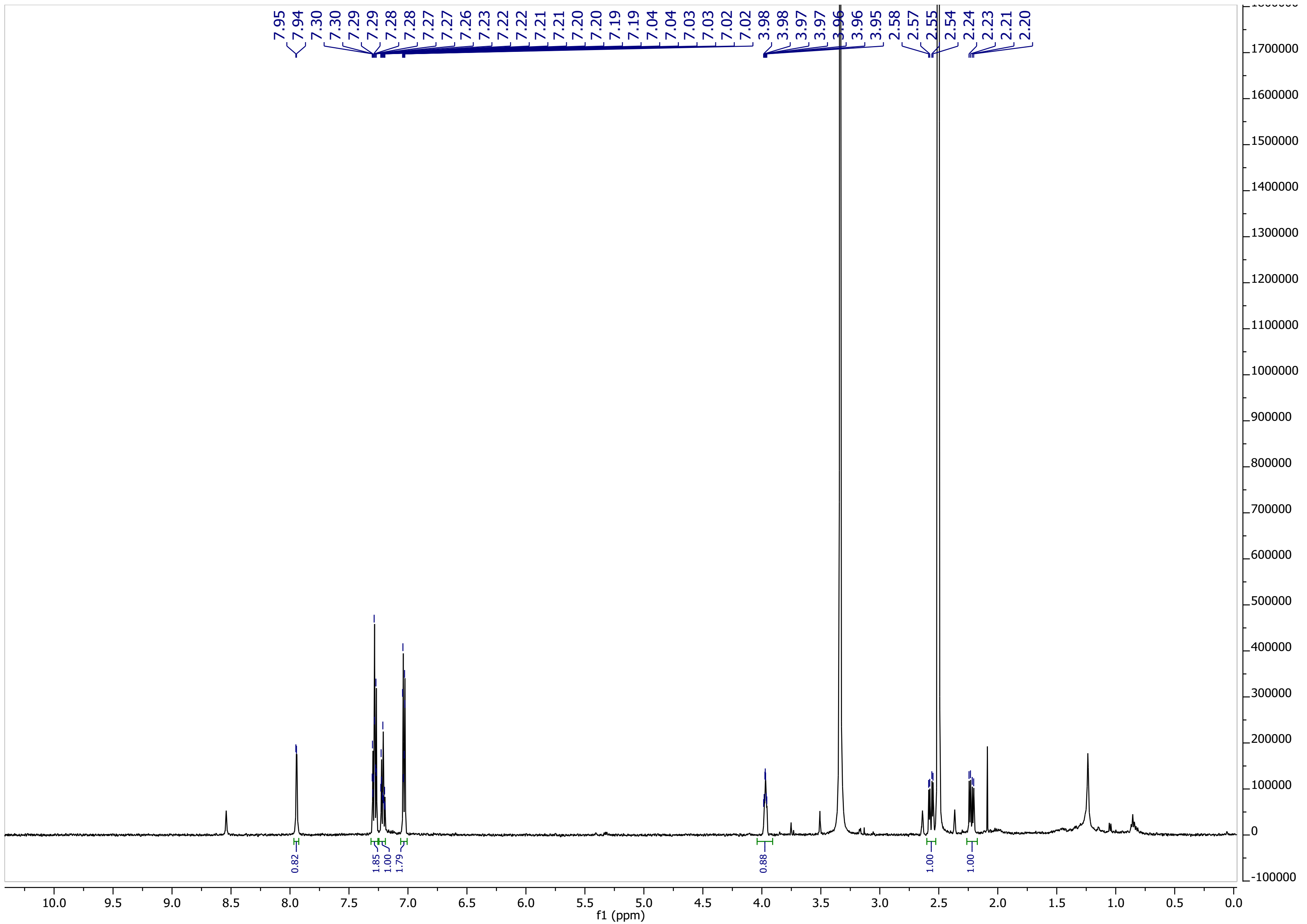

Figure S33. ^{1}H NMR spectrum of **4** in DMSO-d_6 at 700 MHz.

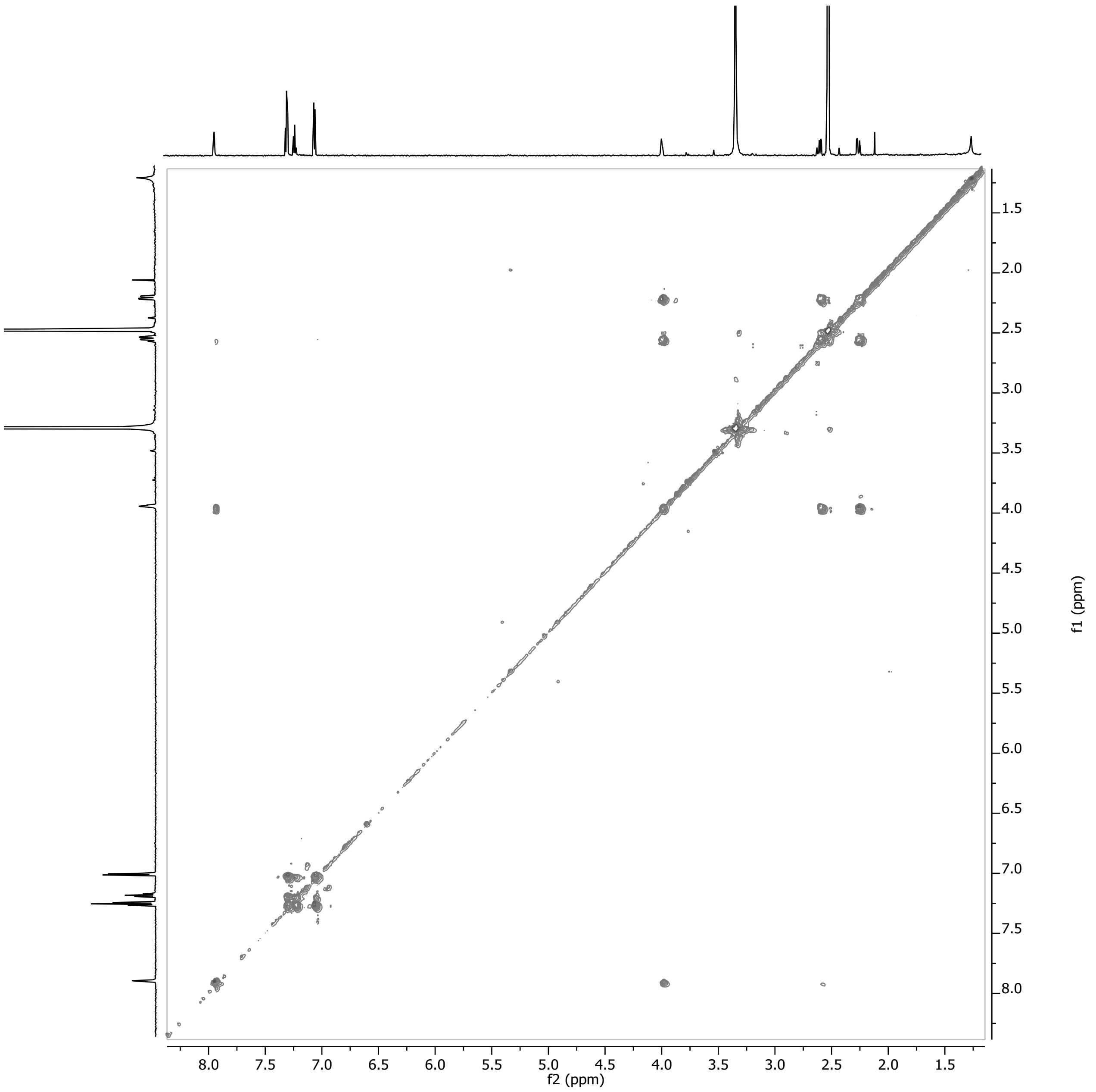

Figure S34. ^{13}C NMR spectrum of **4** in DMSO-d_6 at 175 MHz.

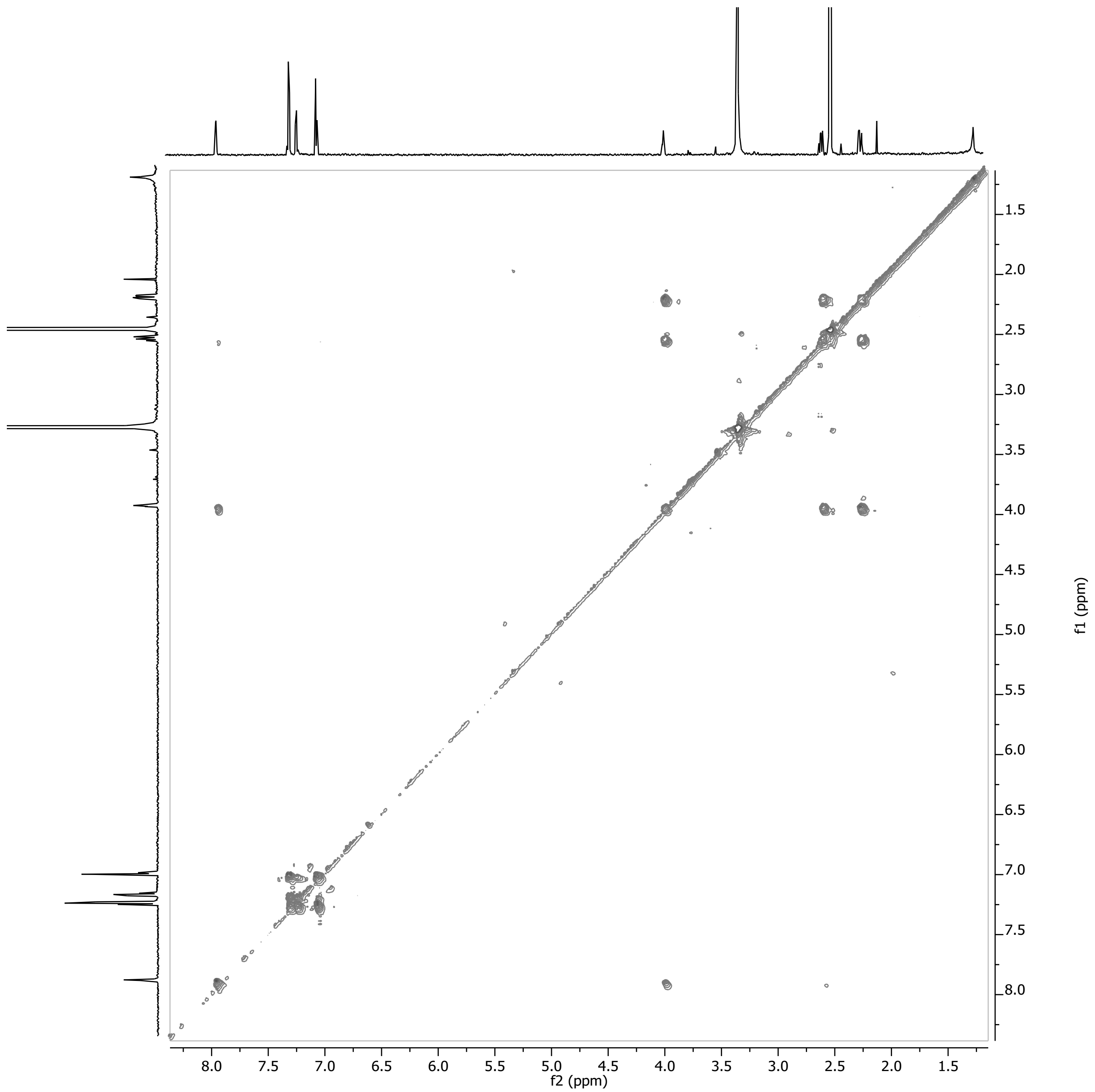

Figure S35. ^{1}H-^{1}H COSY spectrum of **4** in DMSO-d_6 at 700 MHz.

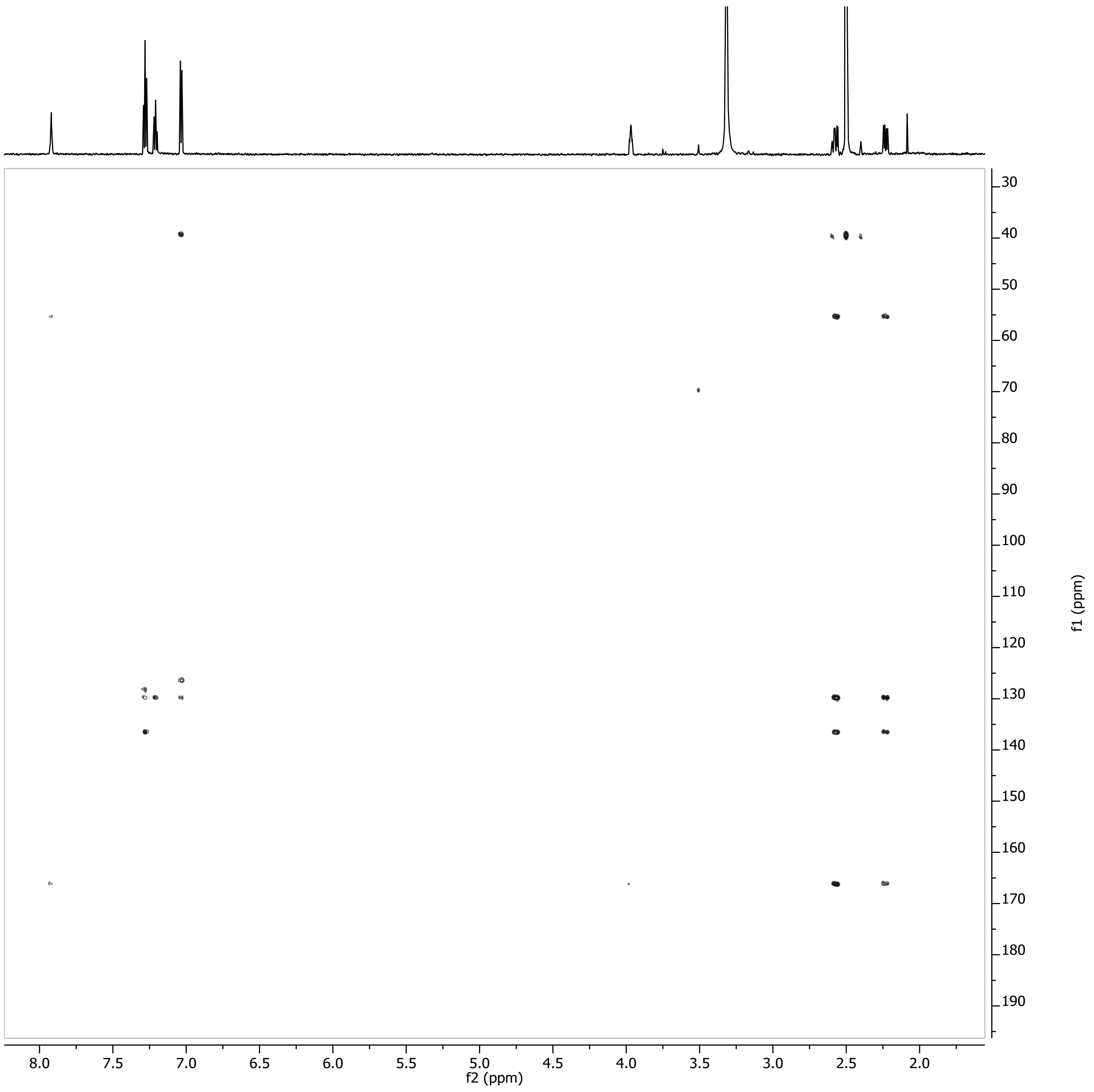

Figure S36. HMBC spectrum of **4** in DMSO-d_6 at 700 MHz.

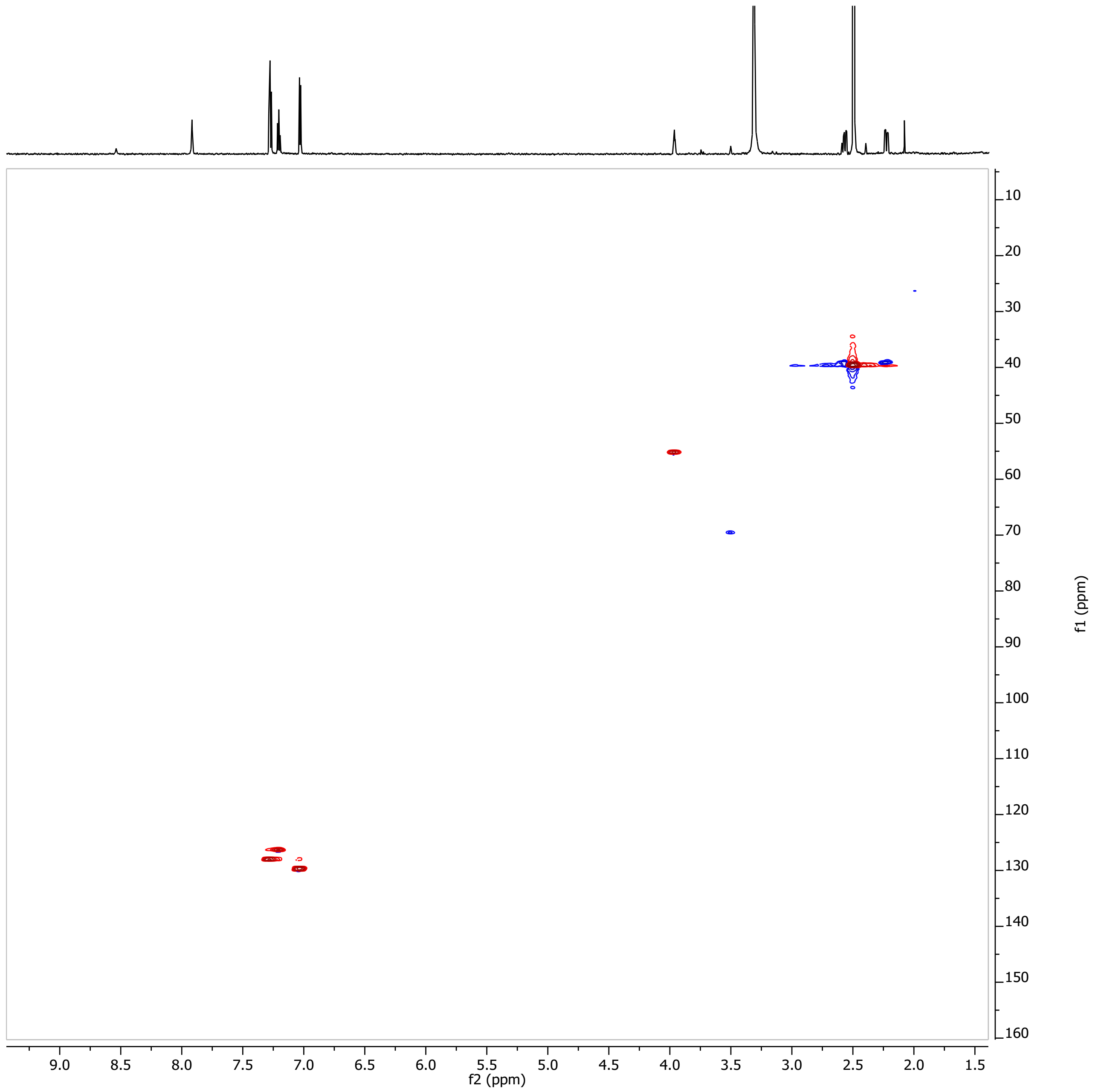

Figure S37. HSQC spectrum of **4** in DMSO-d_6 at 700 MHz.

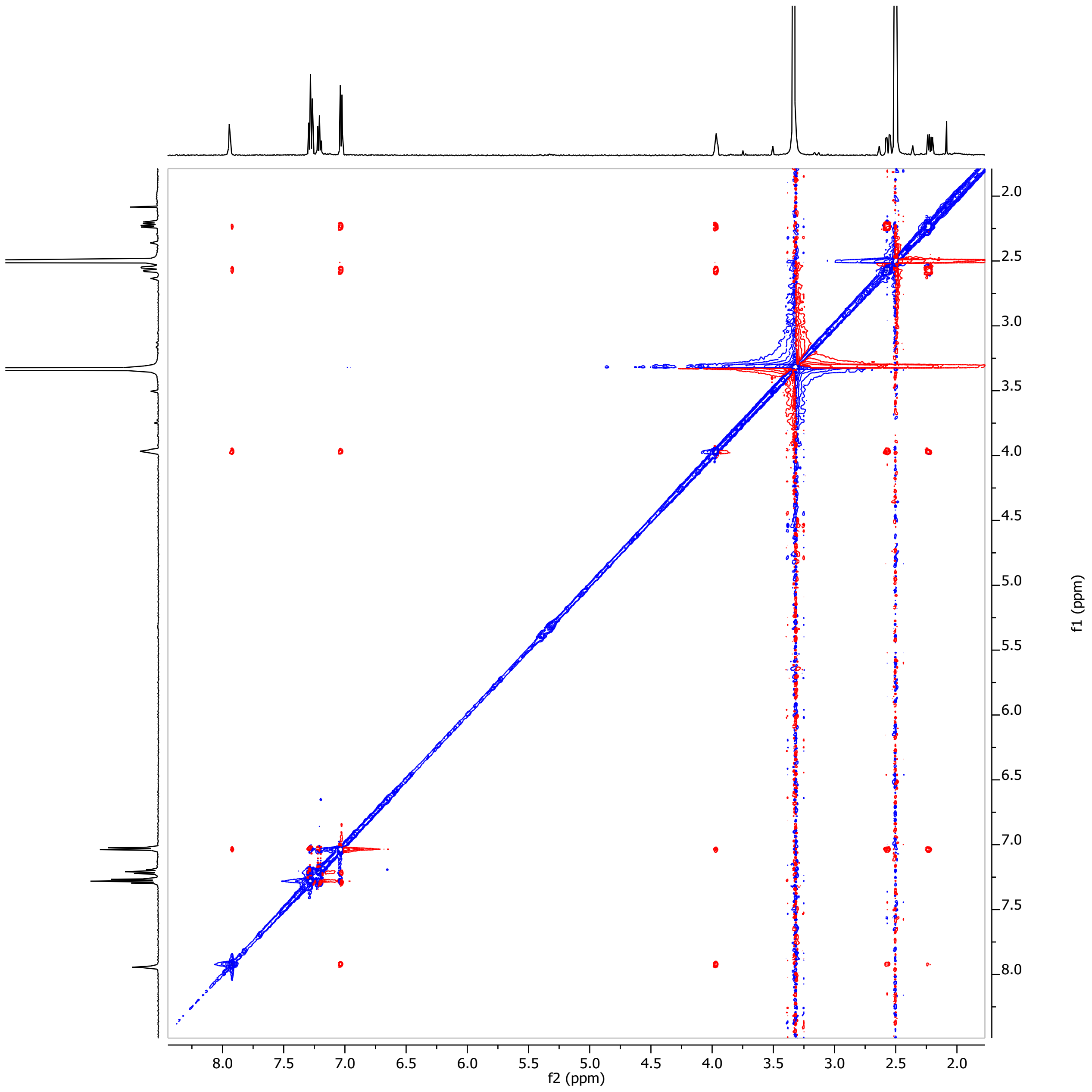

Figure S38. ROESY spectrum of **4** in DMSO-d_6 at 700 MHz.
